# AT-specific DNA visualization revisits the directionality of bacteriophage λ DNA ejection

**DOI:** 10.1093/nar/gkad340

**Published:** 2023-05-09

**Authors:** Serang Bong, Chung Bin Park, Shin-Gyu Cho, Jaeyoung Bae, Natalia Diyah Hapsari, Xuelin Jin, Sujung Heo, Ji-eun Lee, Kaori Hashiya, Toshikazu Bando, Hiroshi Sugiyama, Kwang-Hwan Jung, Bong June Sung, Kyubong Jo

**Affiliations:** Department of Chemistry, Sogang University, Seoul 04107, Korea; Department of Chemistry, Sogang University, Seoul 04107, Korea; Department of Life Science, Sogang University, Seoul 04107, Korea; Department of Chemistry, Sogang University, Seoul 04107, Korea; Department of Chemistry, Sogang University, Seoul 04107, Korea; Chemistry Education Program, Department of Mathematics and Science Education, Sanata Dharma University, Yogyakarta 55282, Indonesia; Department of Chemistry, Sogang University, Seoul 04107, Korea; College of Agriculture, Yanbian University, Yanji133000, China; Department of Chemistry, Sogang University, Seoul 04107, Korea; Department of Life Science, Sogang University, Seoul 04107, Korea; Department of Chemistry, Graduate School of Science, Kyoto University, Sakyo-Ku, Kyoto606-8502, Japan; Department of Chemistry, Graduate School of Science, Kyoto University, Sakyo-Ku, Kyoto606-8502, Japan; Department of Chemistry, Graduate School of Science, Kyoto University, Sakyo-Ku, Kyoto606-8502, Japan; Department of Life Science, Sogang University, Seoul 04107, Korea; Department of Chemistry, Sogang University, Seoul 04107, Korea; Department of Chemistry, Sogang University, Seoul 04107, Korea

## Abstract

In this study, we specifically visualized DNA molecules at their AT base pairs after *in vitro* phage ejection. Our AT-specific visualization revealed that either end of the DNA molecule could be ejected first with a nearly 50% probability. This observation challenges the generally accepted theory of Last In First Out (LIFO), which states that the end of the phage λ DNA that enters the capsid last during phage packaging is the first to be ejected, and that both ends of the DNA are unable to move within the extremely condensed phage capsid. To support our observations, we conducted computer simulations that revealed that both ends of the DNA molecule are randomized, resulting in the observed near 50% probability. Additionally, we found that the length of the ejected DNA by LIFO was consistently longer than that by First In First Out (FIFO) during *in vitro* phage ejection. Our simulations attributed this difference in length to the stiffness difference of the remaining DNA within the phage capsid. In conclusion, this study demonstrates that a DNA molecule within an extremely dense phage capsid exhibits a degree of mobility, allowing it to switch ends during ejection.

## INTRODUCTION

Bacteriophage λ has been used as a representative model system for the study of molecular biology and biophysics since Lederberg's first discovery (1). A phage λ is composed of a 63 nm icosahedral capsid head (2), and a 150 nm noncontractile tail (3). The diameter of the nanotube in the tubular tail is 4 nm, which is only twice the diameter of double-stranded DNA (4). The tiny capsid contains a linear DNA molecule comprising 48502 base pairs (5). This virus packs DNA unidirectionally from the left-hand end to the right-hand end of the genome (6–8). A terminase, an ATP-consuming motor protein, binds the left ends of the viral λ DNA concatemer in *Escherichia coli* host cells and docks an empty viral procapsid to insert the DNA through ATP hydrolysis until it cleaves the next cos site (9). Such DNA packaging requires an extremely high force (51 pN) to compress the long genomic DNA molecule into a nanometer space (10,11). Tight packaging leads to strong repulsive interactions due to the negative charges on the DNA backbone, and bending stress due to the persistence length (50 nm), resulting in internal capsid pressures reaching 25 atm (12). This accumulated pressure in these tiny capsids is a fundamental force inducing viruses to eject the DNA genomes into the host cells during infection (13–16).

When considering unidirectional packaging, a question arises which end ejects first? To date, most review papers have stated that the last DNA end packaged would be the first end out, ‘Last In First Out (LIFO)’ (17). Three independent experimental studies supported the LIFO model (18–20). All of them used electron microscopic images of tail proteins cross-linked with the last-packaged DNA end (the right end of the λ genome). They determined the right-left directionality by DNA melting maps since the right end of the λ DNA had an AT-rich region that could melt easily with increased temperature. Most bacteriophages are known to eject from the last packaged end (LIFO), except bacteriophage T4, which follows ‘First In First Out (FIFO)’ with the aid of a protein (18,21–25). LIFO seems reasonable because the capsid is extremely crowded with a near-crystalline density, which was determined by X-ray diffraction and cryo-electron microscopy. For example, Earnshaw and Harrison analyzed diffraction patterns from phage λ viral capsids to show 2.3–2.6 nm DNA–DNA interstrand spacings (26). This spacing matches the 2.4 nm DNA–DNA interstrand distance of the B-form DNA crystal reported by Franklin and Gosling (27). Using cryo-electron microscopy, Lepault et al. visualized a DNA nematic liquid crystal structure in a capsid (28). Cerriteli *et al.* reconstructed a cryo-EM-based three-dimensional spool structure to explain the 2.5 nm spacing resolved by the X-ray diffraction pattern (29). Therefore, it is expected that encapsulated DNA in a mature viral capsid may be completely trapped in a glassy state, resulting in packed ultraslow mobility (30). Accordingly, LIFO is considered a standard model for phage λ ejection.

However, there have been contradictory observations reported in regard to this matter. Before three EM observations of a cross-linked tail protein and the right end of DNA were reported (18–20), Sharp et al. published a paper titled ‘Lack of polarity of DNA injection by Escherichia coli phage λ’ in 1971 (31). They used X-rays to break the DNA in the capsid and then infected *E. coli* host cells with the X-ray-irradiated λ phages. Their analysis of the ejected fragments containing phage DNA ends showed that half of the λ phages ejected their genomic DNA from the left end and the other half ejected their DNA from the right end. This result may be explained if the first entered left end can move relatively freely within the capsid.

Notably, Liu et al. reported a fluid-like disordered core in the center of a crystal-like spooled configuration of stacked DNA (32). The DNA in the capsid had a toroidal spool structure with near crystalline density, but it was ordered in concentric layers in the periphery of the capsid. A stiff DNA polymer with a persistence length of 50 nm should have a relatively low density in the center of the DNA spool in a 63 nm capsid. Evilevitch and his colleagues reported a series of papers regarding the solid-to-fluid transition in the core of a capsid that contained a cholesteric packing pattern (32–35). Their characterization of the fluidic structure of the capsid core demonstrated different mobility regions in the phage capsid. A part of the DNA should be packed in the liquid crystal spool, but the other part of the DNA may move relatively freely within the fluidic core. Evilevitch suggested a fluid-like DNA structure in the capsid core above 33°C (35). Such a phase transition leads to higher disorder and a significant increase in genome fluidity. From the cryo-EM images, one can estimate the ratio of the disordered core to the phage capsid radius (*d*_0_/*R*_c_). For example, Lander *et al.* showed a cutaway view of the λ capsid, in which *d*_0_ = 19 nm and *R*_c_ = 32 nm (36). De Frutos *et al.* reported that the hexagonal crystal thickness was 11.1 nm for phage λ (37). Walker et al. reported that *d*_0_/*R*_c_ was larger than 0.5 for most bacteriophages (38). Another notable observation was that both ends are located close to each other. For example, Haas *et al.* observed cross-linked DNA fragments between the left ends and the right ends, which implied that both ends were present in the near vicinity (39). Hohn *et al.* suggested a model in which both ends could be located in the vicinity of the portal vertex after DNA packaging (40). Recently, Liu *et al.* reported a cryo-EM structure for the herpesvirus, in which both ends were located near the portal vertex, although it was not a phage λ virus (41). If the left and right ends are closely located in the fluidic disordered core near the portal vertex, either end can eject first because the left end of DNA may not be packed in the crystal. Accordingly, it remains unclear whether phage λ is ejected only from the right end.

In this paper, we visualized the directions of phage λ ejection using AT-specific DNA staining methods that we recently developed (42,43). For *in vitro* phage ejection, we used three induction methods: these methods included the λ phage receptor protein of LamB (44), a cross-linking reagent of glutaraldehyde (45), and a high temperature of 65°C (46). In a microfluidic flow cell, the ejected DNA molecules were immobilized on the surface by an airflow drying method (47) and AT-specific staining. Contrary to the status quo, we found that both ends could eject first, consistent with the result of Sharp *et al.* (31). Interestingly, the ejected DNA lengths were not the same: the ejected DNA lengths of LIFO were consistently longer than those of FIFO. To explain our experimental observations, we performed computer simulations to investigate the process involved in the ejection of DNA with a compact structure. Moreover, our simulations reproduced results consistent with the experimental observation that the ejection lengths of LIFO were longer than those of FIFO.

## MATERIALS AND METHODS

### Chemicals

DNA primers and oligonucleotides were purchased from COSMOGENETH (Seoul, Korea). The fluorescent protein plasmid for mScarlet was purchased from Addgene (Watertown, MA). Neutravidin was purchased from ThermoFischer Scientific (Waltham, MA). Enzymes and λ DNA (48.5 kb) were purchased from New England Biolabs (Ipswich, MA). Tryptone and Luria-Bertani broth (LB) were purchased from Becton, Dickinson and Company (Franklin Lakes, NJ). Low melting point (LMP) agarose was purchased from Invitrogen (Carlsbad, CA), and agarose was from Young Sciences, Inc. (Bucheon, Korea). Epoxy was from Permatex (Solon, OH). *N*-[3-(Trimethoxysilyl)propyl]ethylenediamine was purchased from Acros organics (Fair Town, NJ). Ni-NTA agarose resin and disposable column (empty gravity column) were purchased from Qiagen (Hilden, Germany). Biotin-PEG-succinimidyl carbonate and PEG-succinimidyl valerate were purchased from Laysan Bio Inc (Arab, AL). Sodium chloride, EDTA, and glacial acetic acid were purchased from Duksan (Ansan, Korea). H_2_O_2_, H_2_SO_4_ and 99.8% ethanol were purchased from JIN Chemical (Hwaseong, Korea). *n*-Dodecyl-β-d-maltopyranoside (DDM) were purchased from Goldbio (St. Louis, MO). *N*-Octyl-β-maltopyranoside (OM) were purchased from Anatrace (Maumee, OH). Glutaraldehyde, sodium bicarbonate, thiamine HCl, and other chemicals were purchased from Merck (Darmstadt, Germany).

### Bacteriophage λ preparation

Bacteriophage λ (λBP5203) was obtained from ATCC. The phage λ preparation procedure was based on a previous study (48). Briefly, *E. coli* K-12 MG1655 was grown in 5 ml of LB solution to reach an optical density of 0.6 at 600 nm (OD_600_). Then 1 μl bacteriophage λ solution was mixed with 200 μl LB solution containing *E. coli* cells. This solution was mixed with LMP agarose containing TNT media (3.0 g tryptone, 1.5 g NaCl, 0.3 mg thiamine HCl, 1.5 g LMP agarose in 300 ml H_2_O). This medium was autoclaved and cooled to 37°C before use. The mixture solution was poured onto an LB agar plate (2.5 g LB broth and 1.5 g agar in 100 ml H_2_O poured into Petri dishes) and placed into a 4°C refrigerator for 30 min. After gelling the mixture solution in the agar plates, the plates were moved into a 37°C incubator overnight. Then, 5 ml of LB solution was poured onto the agar plates and placed at room temperature for 1 h. Next, the solution was transferred to 50 ml conical tubes and centrifuged at 4500 rpm for 20 min. Finally, the supernatants from centrifugation were filtered using 200 nm pore syringe filters to obtain phage λ stock solutions.

### LamB preparation

LamB (maltoporin) was prepared as described in Supplementary Information **(**SI). Supplementary Figure S1 illustrates the LamB purification procedure (49,50). The *Lamb* gene (accession number: NP_418460.1) was isolated from the *E. coli* UT5600, cloned and expressed. The resulting LamB protein was purified and stored at −20°C. A more detailed description of the cloning, expression, and purification methods can be found in the Supplementary information Materials and Methods section.

### ATTO647N-β_2_-py_4_-β-py_4_-dp (AP8) synthesis

A computer-assisted Fmoc solid-phase synthesis was performed to produce H_2_N-P8 (1.3 mg, 1.0 × 10^−3^ mmol), as previously described (42). ATTO647N was then added to the N-terminus using ATTO647N NHS ester. After the reaction was completed, the resulting product was purified by high-performance liquid chromatography (HPLC), and the collected fractions were lyophilized to obtain AP8 as a blue powder. A more detailed description of the synthesis and characterization of AP8 can be found in the Supplementary Information (SI) Materials and Methods section, along with SI Supplementary Figures S2∼S4.

### H-NS-mScarlet preparation

Histone-like nucleoid-structural protein (H-NS) fused with mScarlet was prepared as previously described for H-NS-mCherry (43). To enhance the brightness, mCherry (*εϕ* = 15.84 mM^−1^ cm^−1^) was replaced with mScarlet (*εϕ* = 70.0 mM^−1^ cm^−1^) (51). The plasmid pET-15b was used to construct H-NS-mScarlet by adding the PCR-amplified mScarlet fragment to the C-terminus of H-NS. A more detailed description of the construction, expression, purification, and characterization methods can be found in the Supplementary Information (SI) Materials and Methods section, along with Supplementary Figures S5 and S6.

### Microscopy

The microscope system consisted of an inverted microscope (Olympus IX70, Tokyo, Japan) equipped with a 100× Olympus UPlanSApo oil immersion objective lens and illuminated LED light source (SOLA SM II light engine, Lumencor, Beaverton, OR). The light was passed through the corresponding filter sets (Semrock, Rochester, NY) to excite the fluorescent dye. Fluorescence images were captured using a scientific-grade complementary metal-oxide-semiconductor digital camera (2048 × 2048, Prime sCMOS Camera, Photometrics, Tucson, AZ) and stored in 16-bit TIFF format as generated by the Micro-manager software. ImageJ was utilized for image processing and measuring the lengths of ejected DNA molecules.

### The PEG-biotin-coated glass surface preparation

The PEG-biotin-surface was prepared as described in our previous study (52). Briefly, glass coverslips were stacked in a Teflon rack and soaked in a piranha etching solution (30:70 v/v H_2_O_2_/H_2_SO_4_) for 2 h. Then, the glass coverslips were rinsed with deionized water until the pH was neutral (pH 7), followed by sonication for 30 min. The cleaned coverslips were placed in a polypropylene container into which 200 ml of methyl alcohol was added. Then, 2 ml of *N*-[3-(trimethoxysilyl)propyl]ethylenediamine and 10 ml of glacial acetic acid were added. This container was shaken at room temperature for 30 min, sonicated at 75 W for 15 min, and then shaken overnight at 100 rpm at room temperature. Then, the coverslips were washed once with methyl alcohol and twice with ethyl alcohol. For biotinylation, 50 μl of PEG-biotin solution was dropped onto a glass coverslip, covered with PEG-biotin for 3 h, and then rinsed with water. The PEG-biotin solution was prepared as 350 μl containing 0.1 M sodium bicarbonate with 80 mg of mPEG-succinimidyl valerate and 2 mg of biotin-PEG-succinimidyl carbonate.

### Flow cell preparation

**Figure 1.**
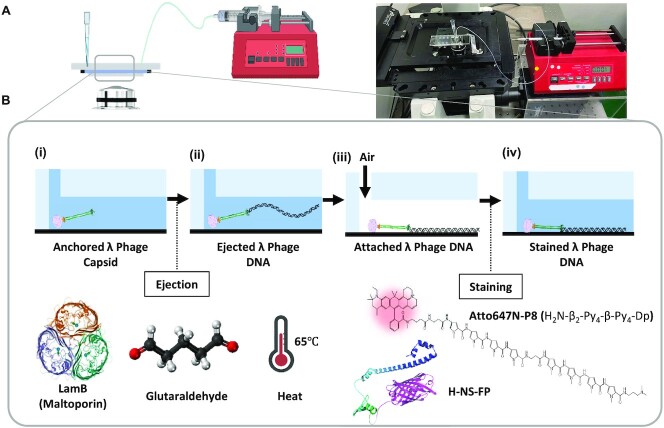
Scheme for phage λ ejection experiment. (**A**) Schematic and image of a flow-cell (100 μm × 1 mm × 10 mm = 1 μl) set up on a microscope controlled by a syringe pump. (**B**) Experimental procedure: (i) Loading phage λ particles to anchor on a PEG-biotin-neutravidin coated surface in a chamber. (ii) LamB, glutaraldehyde, and 65°C treatments to induce phage ejection. (iii) Air drying for DNA immobilization onto the surface. (iv) Staining of ejected DNA with AP8 (Atto647N-octapyrroles) or H-NS-FP. The PDB file for H-NS-mScarlet was created using Alphafold2 (78). (©Biorender-biorender.com).

Figure 1A shows the flow cell set up on a fluorescence microscope. The flow chamber was fabricated by placing an acrylic support on a PEG-biotin-coated glass coverslip with a height of 100 μm, which was formed by a double-sided adhesive sheet as previously described (52). The flow chamber dimensions were approximately 5 × 10 × 0.1 mm (*L* × *W* × *H*). The channels were made with laser cutting. A yellow pipette tip was attached to the inlet port, and a tubing (inner diameter: 0.02 in.) was epoxidized to holes on the outlet port of an acrylic holder. The outlet port was connected to a NE-1000 syringe pump (New Era Pump Systems Inc., Wantagh, NY) with a flow rate of 100 μl/min. Before each experiment, the PEG-biotin-coated glass coverslip was coated with neutravidin. Next, 20 μg/ml of neutravidin in T50 buffer (10 mM Tris, 50 mM NaCl, pH 8.0) was added into a chamber, followed by incubation at room temperature for 5 min.

### Phage ejection visualization

λBP5203 (2.5 μl) was incubated with 0.5 μl of DNase I, and 0.5 μl at 37°C for 15 min to remove prematurely released DNA from the NEB DNase I buffer (total volume = 5 μl). This phage solution was loaded into a flow cell and then incubated for 10 min at room temperature to facilitate phage particles immobilization on the PEG-biotin-neutravidin-coated surface. To remove DNase I, the flow cell was washed with 300 μl of 1× TE buffer with a pH of 8.0 (10 mM Tris, 1 mM EDTA). For phage ejection, phage receptor protein, 25 μg/ml of LamB diluted with NTE buffer (150 mM NaCl, 40 mM Tris–HCl at pH 8.0, 1 mM Na_2_-EDTA, 1% *n*-octyl-β-maltopyranoside (OM), 1 mM DTT, and 30% glycerol), was loaded into the chamber. After 5 min of incubation, individual DNA was ejected. In the second method, phage particles were incubated with glutaraldehyde for 15 min to eject DNA from the capsid. The loaded glutaraldehyde concentration was 2.5% in 0.2 M sodium phosphate buffer (pH 7.25). In the third method, a high temperature was used to eject DNA from the capsid (35). Phage particles were incubated for 5 min at 65°C. For microscopic visualization, H-NS-mScarlet or AP8 in 1× TE buffer was added to stain the ejected DNA molecules. For sequence-specific visualization, the ejected and immobilized DNA should be incubated with H-NS-mScarlet or AP8 for at least 15 min. Alternatively, Supplementary Movie S1 shows the real-time observation of phage ejection stained by YOYO-1. This is based on the fact that YOYO-1 staining is fast, and YOYO-1 is small enough to penetrate the phage capsid. More information can be found in the Supplementary Information (SI) Materials and Methods section, along with Supplementary Movie S1.

### Langevin dynamics simulation

For the DNA simulations, we employed a generic coarse-grained model of a semiflexible chain composed of 200 monomers of diameter σ and mass m. We performed Langevin dynamics simulations with a velocity-Verlet integrator and an integration time step of 0.005$\tau$, where $\tau$ is the reduced time unit, $\tau \equiv \sqrt {m{\sigma }^2/{k}_BT}$. The chemical bond between two neighboring monomers was described by the finite-extensible nonlinear elastic potential (U_b_),


$$\begin{eqnarray*}{U}_b = - \frac{1}{2}KR_0^2ln\left[ {1 - {{\left( {\frac{r}{{{R}_0}}} \right)}}^2} \right] + 4\epsilon \left[ {{{\left( {\frac{\sigma }{r}} \right)}}^{12} - {{\left( {\frac{\sigma }{r}} \right)}}^6} \right] + \epsilon ,\end{eqnarray*}$$

where *K* = 30*k_B_T*/σ^2^ and *R*_0_ = 1.5σ as in the standard polymer model of Kremer and Grest. $\epsilon$ = *k_B_T* is the unit of energy, where *k*_*B*_ and *T* denote the Boltzmann constant and temperature, respectively. The nonbonding interactions between monomers were described by the repulsive Weeks–Chandler–Andersen (WCA) potential. We also imposed a bending potential (*U_a_*) between two consecutive chemical bonds, i.e. *U_a_* = ${K}_a$ (1 + cos θ), where θ is the angle between two neighboring bonds. We set σ = 5 nm and ${K}_a$ = 10*k_B_T* such that the persistence length (*l*_*p*_) of our polymer chain was comparable to *l*_*p*_ = 10σ = 50 nm of a double-stranded DNA. To investigate the effects of the rigidity differences (persistence length) between the first and the second halves of the λ DNA, we assigned the different values of *K_a_* to the first and the second halves, i.e. ${K}_F$ and ${K}_L$, respectively. We define the relative rigidity as $r\ = \ ( {{K}_F - {K}_L} )/{K}_L$ and changes the value of $r$ from 0 to 0.35 by setting (${K}_F$, ${K}_L$) = (10, 10), (10.5, 9.5), (11, 9) and (11.5, 8.5). Note that the average values of ${K}_F$ and ${K}_L$ were equal to 10*k_B_T* in all cases.

The spherical capsid was composed of 784 particles. We considered capsids of two sizes such that the packing fraction of the capsid (when the DNA is fully packaged) was either 0.4 or 0.6. When the packing fraction was as high as 0.6, the DNA was packed inside the capsid such that the conformational relaxation was quite slow. We attached a cubic box of 1.6σ × 1.6σ × 10σ as a portal to the capsid. We prepared 2000 initial configurations for each condition by placing the first 10 monomers of the DNA inside the portal and adding 190 monomers sequentially at random positions outside the portal. We followed three steps to mimic the ejection process: (i) packaging, (ii) resting and (iii) ejection. In the packaging step, we applied an external packaging force (${f}_p$) to the monomers inside the portal and package monomers into the capsid. The packaging force generated by the motor protein of λ phage was approximately 85 pN (53), which corresponds to approximately 8*k_B_T*/σ in this simulation model. In this work, we employed a packaging force of 10*k_B_T*/σ, which was slightly larger than that of λ phages. Then, we adopted a stalling step during the packaging process to mimic the packaging process in the viral capsid. Every time 50 monomers were packaged, we turned off the packaging force, fixed 10 monomers inside the portal at their positions and allowed the other parts of the DNA to relax their conformation during ${\tau }_{stall}$ = 500$\tau$. After the packaging was completed with all 200 monomers inside the capsid, we blocked the entrance of the portal. Then, we initiated the resting step where the DNA chain inside the capsid was allowed to undergo conformational relaxation during the resting time (${\tau }_{rest}$). We changed the value of ${\tau }_{rest}$ from 0 to 1500$\tau$ to investigate the effects of conformational relaxation on the directionality of DNA ejection. Once the resting step was completed, we eliminated the blockage, introduced a capsid portal, and let the DNA spontaneously eject from the capsid.

In the ejection step, we introduced the resisting force (${f}_r = \ 0.35\ {k}_BT/{\rm{\sigma }}$) at the end of the capsid portal to mimic the effect of the osmotic pressure outside the capsid. In the *in vitro* experiments (12–56), the whole part of the DNA did not eject, and a fraction of monomers remained inside the capsid due to the resisting force induced by the osmotic pressure. When we applied a resisting force of ${f}_r = \ 0.35\ {k}_BT/{\rm{\sigma }}$, 30% of the DNA did not eject from the capsid even after a sufficiently long time in the simulations, which is comparable to that in experiments. We checked the index (*i_exit_*) of the monomer ejected from the capsid for the first time. The probability of FIFO was estimated out of 2000 independent trajectories.

## RESULTS AND DISCUSSION

Figure 1 illustrates the design of a single-molecule experiment using a fluorescence microscope to determine the orientation of DNA ejected from a phage capsid. The flow cell setup shown in Figure 1A was the primary platform for performing multistep reactions by loading reagents through simply changing the pipette tips (52). Before loading phage particles into the flow cell, DNase I was used to remove prematurely released DNA molecules without induction. The loaded λ capsid was bound to the surface (Figure 1B (i)) coated with PEG-biotin-neutravidin. Three different methods were used for *in vitro* phage ejection: these methods included LamB (44), glutaraldehyde (45) and 65°C heating (46) (Figure 1B (ii)). All three induction methods were successful for phage λ ejection. First, we used LamB since most *in vitro* phage λ ejection experiments have used this protein (57). LamB is a maltose outer-membrane porin and acts as a bacteriophage λ receptor in host *E. coli* cell membranes (58). Thus, we constructed an expression vector to obtain the LamB protein as described in the methods section (SI Methods and Supplementary Figure S1). Second, we used the fixation chemical glutaraldehyde because the fixation-induced phage ejection method has been used in many studies, such as those regarding T7 (45), T4 (59) and SPP1 (23). Although the detailed mechanism is still unknown, Kellermayer et al. suggested that the process may involve the lever-like action of the tail fibers to cause a transition within the tail complex (60). The SI Movie S1 illustrates the real-time phage ejection process using glutaraldehyde induction and YOYO-1 staining. Third, we used 65°C heating, which was based on previous studies that showed that DNA could be ejected at a temperature between 50°C and 75°C (46). When optimizing the experimental conditions, the heat-induced ejection method was mainly used because it was simple, convenient, and compatible with subsequent procedures in terms of cleanliness. The DNA molecules ejected from phage capsids were fully elongated by a syringe pump-driven flow (100 μl/min). Then, the DNA molecules were immobilized on the surface by airflow (47). Finally, the DNA molecules were stained with AT-specific ATTO647N-labeled octameric pyrroles (AP8) or AT-specific DNA binding fluorescent protein (H-NS-mScarlet). The detailed characterization information for the AP8 and H-NS-mScarlet can be found in the SI Supplementary Figures S2–S6. These figures provide data such as the synthesis scheme and molecular weight determined by mass spectrum (Supplementary Figure S2), binding affinity against AT0 (GC-only), AT4, AT7 and AT10 DNA oligomers determined by fluorescence resonance energy transfer (FRET) for AP8 (Supplementary Figure S4), and SDS-PAGE and gel filtration chromatography for H-NS-mScarlet (Supplementary Figure S5), as well as its binding affinity determined by FRET (Supplementary Figure S6). In a previous study, we reported the development of tetramethyl rhodamine (TAMRA)-labeled octameric pyrroles (TP8) for AT-specific DNA staining (42). However, since TAMRA has low brightness (*εϕ* = 18.1 mM^−1^ cm^−1^), in this study we substituted it with the much brighter ATTO647N (*εϕ* = 97.5 mM^−1^ cm^−1^) (61,62). The SI Supplementary Figure S3 provides a comparison of the brightness levels between AP8 and TP8. Similarly, we substituted H-NS-mCherry (*εϕ* = 15.84 mM^−1^ cm^−1^) (43) with H-NS-mScarlet (*εϕ* = 70.0 mM^−1^ cm^−1^) (51,63). The size exclusion chromatograph shown in SI Supplementary Figure S5 demonstrates that H-NS-mScarlet forms multimers or aggregates.

**Figure 2.**
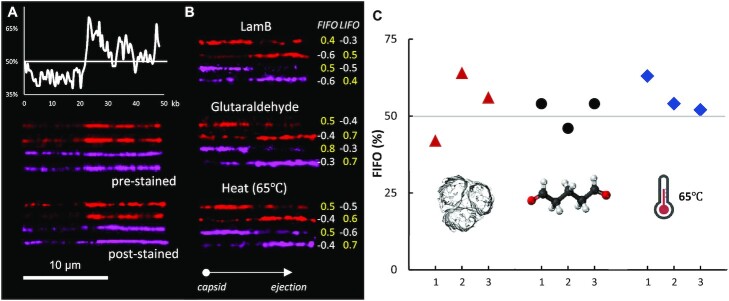
AT-specific staining to identify the directionality of phage λ ejection. (**A**) AT frequency profile for λ DNA (48.5 kb). The Y-axis indicates the percentage of AT content. Single-molecule images of AT-specifically stained λ DNA obtained from NEB with AP8 (8 μM) and H-NS-mScarlet (20 nM). Prestained DNA molecules were stained in a test tube and poststained DNA molecules were stained in the flow cell after air-drying immobilization. For poststaining, diluted AP8 (27 nM) was used. (**B**) Single-molecule images of ejected phage λ DNA after the three induction methods (LamB, glutaraldehyde, 65°C heating) to represent FIFO (First In First Out) and LIFO (Last In First Out). The red DNA was stained with AP8 (27 nM) and the pink DNA was stained with H-NS-mScarlet (20 nM). DNA orientation was determined by Pearson cross-correlation coefficients, with the capsid being oriented to the left and the ejected DNA being oriented to the right. The positive coefficients in yellow indicate the direction of phage ejection. (**C**) Percentage of FIFO using LamB (red triangle, 54 ± 9.1%), glutaraldehyde (black circle, 51 ± 3.8%), and 65°C heating (blue diamond, 56 ± 4.8%). Each condition had three sets, two stained by AP8 and one stained by H-NS-mScarlet. Each set contained 50 phage ejection data.

Figure 2 demonstrates how to identify FIFO and LIFO occurrences based on DNA images. The λ genome contains a GC-rich (AT-poor: AT content less than 50%) region of up to 20 kb and an AT-rich (AT content more than 50%) region of 20–48.5 kb with some variations (Figure 2A). The AP8-stained red λ DNA images matched well to reveal detailed intensity profiles for AT abundance. The subsequent H-NS-mScarlet-stained pink DNA images were also good enough to determine the directionality of the λ DNA molecules, although they were noisier than the AP8-stained DNA images, likely due to the multimeric aggregations of H-NS-mScarlet. These four DNA images were produced by a prestaining procedure in which λ DNA was incubated with AP8 or H-NS-mScarlet in a test tube for 15 min and then loaded into the flow cell. However, to apply the AT-specific staining method in the *in vitro* phage ejection experiments, it was necessary to consider poststaining because the phage should eject its DNA into the flow cell before staining. Therefore, as a control, we acquired the next four DNA images using a poststaining procedure. First, biotin-end labeled λ DNA molecules were loaded and tethered to a neutravidin-coated surface. Second, DNA was immobilized on the surface with airflow. Finally, the staining reagent solution was loaded and incubated for at least 15 min. For the poststaining procedure, we had to reduce the AP8 concentration from 8 μM to 27 nM, because 8 μM was too high for poststaining in a flow cell, as it generated strong background noise. The DNA image quality obtained from the poststaining procedure was not as good as that obtained from the prestaining procedure. However, the orientation of the λ DNA was recognizable by the thin line demonstrated by the AT-poor region up to 20 kb and the thick line demonstrated by the AT-rich region after 20 kb.

Using this AT-specific staining, we performed single-molecule DNA observations to determine the DNA ejection directionality, i.e. FIFO versus LIFO, during phage λ ejection. It would be ideal to capture a movie of phage ejection using AT-specific staining reagents, but it is not possible due to the mismatch between the time required for phage ejection (a few seconds, as shown in SI Movie S1) and the incubation time for AT-specific staining (at least 15 min), which does not allow for the creation of a movie. Figure 2B shows examples of FIFO and LIFO induced by the three ejection methods. In each case, the first two images were of DNA stained with AP8, and the last two images were of DNA stained with H-NS-mScarlet. We calculated Pearson cross-correlation coefficients to determine the direction of DNA. The positive and negative coefficients clearly determine FIFO or LIFO. We performed three sets of experiments for each induction method, and each set had 50 ejected DNA images. Figure 2C shows the percentage of FIFO (LIFO = 100 − FIFO). The results of both the FIFO and LIFO percentages were approximately 50% for all data sets. Specifically, the FIFO percentage was 53.9 ± 7.0% and the LIFO percentage was 46.1 ± 7.0%.

To validate our single-molecule observations using an alternative method, we designed an experiment as shown in SI Supplementary Figure S7. Three methods, such as LamB, glutaraldehyde, and 65°C, were used to induce DNA ejection from the capsid, followed by digestion with DNase to degrade any ejected DNA. After inactivating the DNase, we performed qPCR using five primers targeting fragments at 0.5, 12.1 24.1, 36.2 and 47.7 kb. The PCR process involves heating the sample to 95°C, which disrupts the phage capsid and releases the DNA remaining in the capsid for amplification. The resulting cycle threshold (Ct) values in SI Supplementary Figure S7 are the same in both ends and high at 24.1 kb, meaning that the 24.1 kb fragment had a much lower abundance compared to the others. Specifically, for the case of LamB, the Ct values were 13.33 (0.5 kb), 12.62 (12.1 kb), 28.92 (24.1 kb), 13.74 (36.2 kb) and 13.22 (47.7 kb) respectively. To establish a control, we conducted the same qPCR experiment using NEB lambda (500 pg in 20 μl). The resulting CT values were 11.3 (0.5 kb), 10.94 (12.1 kb), 10.9 (24.1 kb), 11.89 (36.2 kb) and 11.43 (47.7 kb), respectively, which were even for all positions. Therefore, we can interpret these qPCR results as both ends of DNA remained after ejection at the same ratio, but the middle (24.1 kb) was mostly in the ejected DNA and degraded by DNase. These results support our single-molecule observations for both the FIFO and LIFO.

**Figure 3.**
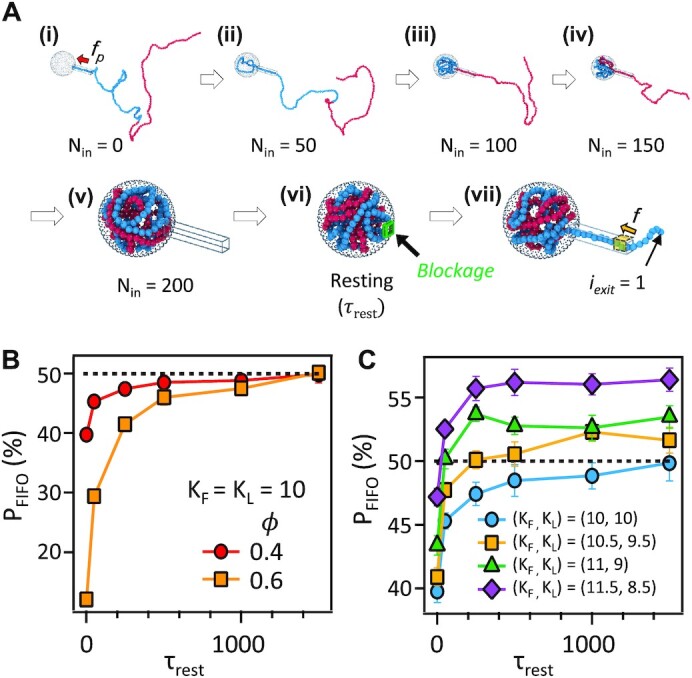
Computer simulation of DNA ejection. (**A**) Simulation snapshots of a representative trajectory for the packaging, resting and ejection steps. Blue and red particles represent the first half and the second half of the polymer chain, respectively. N_in_ denotes the number of monomers that are packaged into the capsid. (i)–(v) The packaging step where the polymer chain is actively packaged into the capsid with the packaging force (${f}_p$ = 10 *k_B_T*/σ) applied to the monomers inside the capsid portal. Every time 50 monomers were packaged, we turned off the packaging force, fixed 10 monomers inside the portal at their positions and allowed the other parts of the DNA to relax their conformation during 500$\tau$. (vi) The resting step where the entrance of the portal is blocked by a blockage (the green cubic box) and the polymer chain inside the capsid is allowed to relax its conformation during ${\tau }_{rest}$. (vii) The ejection step where monomers spontaneously eject through the capsid portal. We counted the index (*i_exit_*) of the monomer that ejects for the first time. We applied a resisting force (${f}_r = \ 0.35\ {k}_BT/{\rm{\sigma }}$) to the last two monomers at the end of the capsid portal (yellow shaded area) to mimic the effects of the osmotic pressure outside the capsid. **(B)** Probability (P_FIFO_) that the monomer (that enters the capsid first) ejects first as a function of the resting time (${\tau }_{rest}$) for two values of the maximum packing fraction ($\phi$) of a polymer chain of the relative rigidity $r$ = 0 with all 200 monomers packaged. Red circle and orange square symbols represent the cases for $\phi$ = 0.4 and 0.6, respectively. P_FIFO_ converges to 50% when enough resting time (${\tau }_{rest}$) is given. (**C**) Simulation results of *P*_FIFO_ as a function of the resting time (${\tau }_{rest}$) for different sets of (${K}_F$, ${K}_L$). The simulation scheme is identical (i–vii) while the parameters (${K}_F$ and ${K}_L$) for the angular potential energy are tuned. P_FIFO_ converges to values about 50% as the resting time (${\tau }_{rest}$) increases.

It is curious how 53.9% of the first-packaged left ends could be ejected first by passing through a DNA structure with near crystalline density. To understand such phage ejection at the molecular level, we performed Langevin dynamics simulations (64,65). Our simulations suggested several factors that could determine the DNA ejection directionality: (i) how easily the DNA relaxes its conformation inside the capsid after packaging, (ii) whether the two ends of the DNA are randomized or not after conformational relaxation and (iii) how rigid the segment (to which each end of the DNA belongs) would be. Our simulation scheme consists of three subsequent steps: packaging, resting, and ejection (Figure 3A). The DNA is allowed to relax its conformation during a designated resting time (*τ*_rest_) in the resting step. As shown in Figure 3B, the probability of FIFO (P_FIFO_) increases to 50% with an increase in *τ*_rest_ depending on the packing fraction (*ϕ*) of the capsid when fully packaged. When the DNA is given a sufficiently long resting time of *τ*_rest_ > 1000*τ* for conformational relaxation, *P*_FIFO_ reaches approximately 50%. In the case of short resting times, the last-packaged end is more likely to be near the capsid portal than the first-packaged end. On the other hand, with a sufficiently long resting time, the positions of the two ends of the DNA are randomized such that the two ends are given equal chances to be located near the capsid portal. We observed a similar trend for a smaller packing fraction of *ϕ* = 0.4: *P*_FIFO_ increases and converges to 0.5 as *τ*_rest_ increases. For a smaller packing fraction, *P*_FIFO_ reaches 0.5 even for a shorter resting time because the DNA can relax its conformation more readily in a less densely packed capsid.

We also investigated the effects of a difference in the rigidity of the DNA segments. As shown in Figure 2A, λ DNA has a GC-rich region on the left and an AT-rich region on the right of the genome. As expected, the GC-rich region is more rigid, and the AT-rich region is more flexible due to the number of hydrogen bonds. Chuang et al. reported a 14 nm difference in the persistence lengths depending on GC contents ranging from 30% to 60% (66). In their study, they developed statistical terpolymer models to predict persistence length (${l}_p$) of DNA as follows, ${l}_p = ( {23 + 23\gamma + 26{\gamma }^2} )\ + c$. Here, $\gamma$ and $c$ represent the fraction of GC content and a constant, respectively. The difference in the persistence length between GC-rich and AT-rich regions is, then, $\Delta \ {l}_p = {l}_{p,GC}\ - \ {l}_{p,AT} = \ 23( {{\gamma }_{GC} - {\gamma }_{AT}} ) + 26{( {{\gamma }_{GC} - {\gamma }_{AT}} )}^2$. As shown in Figure 2A, the fractions ($\gamma$) of GC contents of GC-rich and AT-rich region are about 0.53 (${\gamma }_{GC}$) and 0.47 (${\gamma }_{AT}$), respectively. Therefore, $\Delta {l}_p$ between two regions is estimated about 5.9 nm. Because the average persistence length of overall DNA is 50 nm, $0.45{l}_{p,GC} + 0.55\ {l}_{p,AT} = \ 50$ considering that the portions of GC-rich and AT-rich regions are about 0.45 and 0.55 as shown in Figure 2A. Combining two equations, the persistence lengths of GC-rich and AT-rich regions are estimated to be 53 and 47 nm, respectively. To investigate the effect of difference in rigidities of two regions, in this simulation, we set the persistence length of the first half of the DNA (which includes the first-packaged end and is represented by blue particles in Figure 3A) as longer than that of the second half (which includes the last-packaged end and is represented by red particles) by tuning the values of the force constants (*K_F_* and *K_L_*) of the bending potentials. *K_F_* and *K_L_* indicate longer and shorter persistence lengths of the first half (GC-rich region) and the second half (AT-rich region), respectively. As shown in Figure 3C, *P*_FIFO_ after a sufficiently long resting time (*τ*_rest_) depends strongly on the difference in the persistence length (or *K_F_* > *K_L_*). When the first and second segments are equally rigid with *K_F_* = *K_L_* = 10, *P*_FIFO_ converges to approximately 50%. As the first half of the DNA becomes more rigid than the second half with *K_F_* > *K_L_*, *P*_FIFO_ increases slightly beyond 50%, which agrees with our experimental observations of 53.9% for the FIFO (Figure 2C).

**Figure 4.**
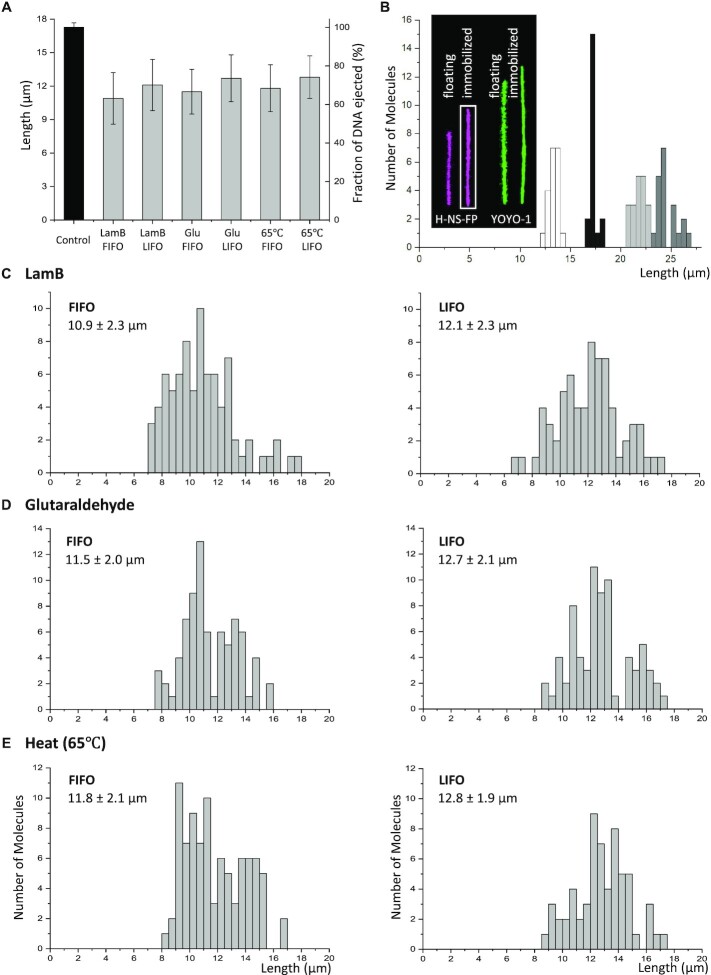
Fractional lengths of ejected phage λ DNA. (**A**) Comparison of ejected phage λ DNA. The black control bar represents the airflow-immobilized λ DNA stained with H-NS-FP in (B). The second y-axis represents the percentage against the control. Error bars represent the standard deviations. (**B**) Control DNA images and their histograms. The images are of tethered and floating (empty bar) and airflow-immobilized (gray bar) λ DNA stained with H-NS-mScarlet (60 nM), and floating and immobilized λ DNA stained with YOYO-1 (1 μM). (C–E) Length distribution of ejected λ DNA by FIFO and LIFO from a phage capsid by the induction methods of LamB (**C**), glutaraldehyde (**D**) and 65°C heating (**E**).

Figure 4 compares the experimentally measured lengths of ejected DNA by FIFO and LIFO in the three induction methods. To estimate the ejection fraction, it is necessary to set the control length to 100%, as shown by the black bar in Figure 4A. To determine the control λ DNA length in this experimental condition, we measured the length of λ DNA purchased from NEB, which was tethered to the surface by the hybridization of a biotin-tagged oligomer and then immobilized on the surface by airflow in the flow cell (47). Figure 4B shows four control histograms and their representative DNA images: 1) H-NS-mScarlet and floating (empty); 2) H-NS-mScarlet and air-immobilized (black bars); 3) YOYO-1 and floating (light gray); 4) YOYO-1 and air-immobilized (dark gray). The black histogram and white boxed DNA image represent the control λ DNA used in Figure 4A. The average length of the control λ DNA was 17.25 ± 0.4 μm, slightly longer than the full contour length (16.5 μm = 0.34 nm/bp × 48 502 bp). Since intercalation dye-stained DNA has been generally used for most single-molecule DNA studies, we also measured YOYO-1-stained DNA. The length of YOYO-1-stained floating λ DNA was 21.8 μm and that of air-immobilized λ DNA was 24.5 μm. According to a previous study showing that the length of YOYO-1-stained DNA could increase by 50% (16.5 μm × 150% = 24.8 μm) (67), 24.5 μm is the value related to the contour length of YOYO-1-stained DNA.

Based on this control length of 17.25 μm, the ejected DNA fractions were 60–75%. It is well-known that *in vitro* phage ejection cannot be complete (68–71). Many *in vitro* phage ejection experiments showed that a fraction of DNA remained in the viral capsid (12,13,44,54–56). When a phage ejects DNA on the membrane of an *E. coli* host cell, osmotic pressure facilitates the completion of phage ejection since the bacterial cytosol has higher osmotic pressure than the extracellular environment. However, *in vitro* DNA ejection cannot be complete because no osmotic pressure gradient forms. Evilevitch et al. demonstrated a gel electrophoresis result of fractional ejections by varying the concentrations of polyethylene glycol (55).

**Figure 5.**
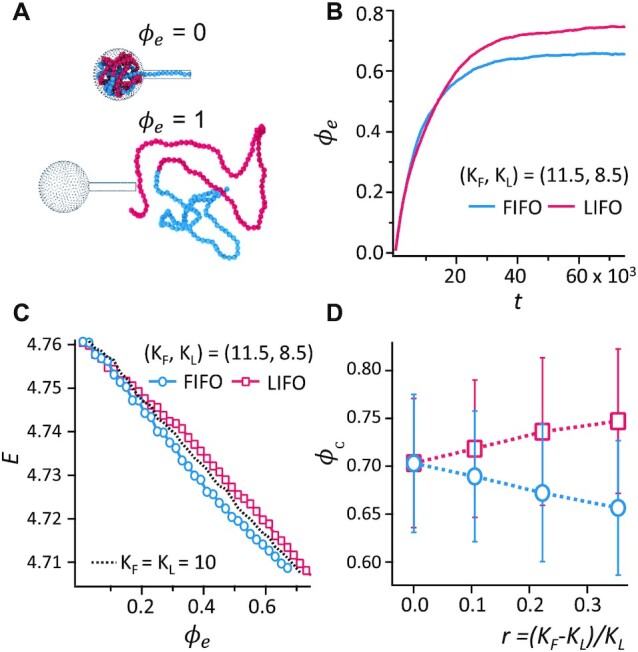
Different ejection fractions via the FIFO and LIFO pathways. (**A**) Simulation snapshots of the ejection step when the ejection fraction (${\phi }_e$) is either 0 or 1. Blue and red particles represent the first and the last halves of the DNA that were packaged first and last, respectively. (**B**) Simulation results of the ejection fraction (${\phi }_e$) as a function of time t by FIFO and LIFO with (${K}_F$, ${K}_L$) = (11.5, 8.5) and ${f}_r$ = 0.35 ${k}_BT/{\rm{\sigma }}$. (**C**) Total energy (*E*) as a function of ${\phi }_e$ during the ejection step. The blue circle and red square symbols correspond to FIFO and LIFO, respectively with (${K}_F$, ${K}_L$) = (11.5, 8.5) and ${f}_r$ = 0.35 ${k}_BT/{\rm{\sigma }}$. A dotted line represents the energy of a polymer chain of ${K}_F$ = ${K}_L$ = 10. (**D**) Simulation results of the converged value (${\phi }_c$) of the ejection fraction (${\phi }_e$) of the FIFO and LIFO pathways.

Figure 4A shows another intriguing fact that the ejected DNA lengths by LIFO were consistently longer than those by FIFO in all three induction methods, although they were within the error ranges. The histograms in Figure 4C–E more clearly show that the ejected fractions of LIFO were longer than those of FIFO. All LIFO histograms peaked at approximately 12–14 μm, but the FIFO histograms peaked at approximately 9–11 μm. To explain our observations, we conducted computer simulations, in which we found a plausible reason that the difference in the rigidity between the first half (GC-rich region) and the second half (AT-rich region) of λ DNA may lead to the difference in the ejected fractions by FIFO and LIFO. In our simulations, we quantified the extent of the ejection process by defining the ejection fraction (${\phi }_e$) as the ratio of the number of ejected monomers to the total number of monomers. As shown in Figure 5A, ${\phi }_e$ = 0 and 1 correspond to the states before the ejection begins and when all the monomers eject, respectively. During the ejection step in our simulations, we introduced a resisting force (${f}_r$) of 0.35 ${k}_BT$/σ, which is representative of the osmotic pressure outside the capsid. This approach allowed us to reproduce the values of ${\phi }_e$ in our experiments (Figure 4A). Due to the resisting force, the ejection fraction (${\phi }_e$) does not reach 1 in our simulations even after a sufficiently long time, thus indicating that a certain fraction of monomers remains inside the capsid. Figure 5B depicts the ejection fraction (${\phi }_e$) as a function of time for $({K}_F$, ${K}_L$) = (11.5, 8.5). As the ejection step begins, ${\phi }_e$ increases but converges to a value (${\phi }_c$) of less than 1. The converged value (${\phi }_c$) of the ejection fraction (${\phi }_e$) depends on whether the ejection proceeds by FIFO or LIFO pathways. When the ejection proceeds by the LIFO pathway, ${\phi }_c\ \cong 0.75$ such that approximately 25% of monomers do not eject. When the DNA proceeds by the FIFO pathway, ${\phi }_c\ \cong 0.66$. Therefore, the fraction of ejected DNA by FIFO is smaller than that by LIFO, consistent with our experimental observation (Figure 4A). This simulation result also explains the results of Supplementary Figure S7, which shows the qPCR results of the ejected DNA degraded by DNase. The qPCR results indicate that λ DNA at 12.1 kb (25% of the λ genome) and 36.2 kb (75% of the λ genome) were more abundant than the middle (24.1 kb) after DNase treatment, consistent with the predicted fractions remaining in the capsid.

A remaining question is why the length of ejected DNA is longer via the LIFO pathway. To answer this question, we investigated how the total energy (*E*, the potential energy plus the kinetic energy) of the DNA changes during the ejection process in our simulations. If the DNA is allowed to relax its conformation for a sufficiently long time, the total energies (*E*) at two limiting states of *ϕ*_e_ = 0 and 1 are independent of whether the ejection occurs via the FIFO pathway or LIFO pathway. However, when the rigidity of the first half (GC-rich region) of the DNA is higher than that of the second half (AT-rich region) (${K}_F >$${K}_L$), the DNA follows a different pathway in the energy landscape during ejection. Figure 5C depicts the total energy (*E*) of the DNA of (${K}_F$, ${K}_L$) = (11.5, 8.5) during the ejection processes. When the first half of the DNA, which is more rigid, ejects first and the more flexible second half of the DNA remains in the capsid (FIFO pathway), the total energy *E* is lower during the ejection process than when the DNA proceeds by the LIFO pathway. This is because the bending energy (which would create a high internal pressure inside the capsid) is relaxed more efficiently via the FIFO pathway. Such a difference in the total energy *E* between the FIFO and LIFO pathways is most prominent in the middle of the ejection process at ${\phi }_e$ = 0.5. Since the total energy is higher along the LIFO pathway, the internal pressure inside the capsid should also be higher when the DNA proceeds by the LIFO pathway instead of the FIFO pathway. This makes the ejection of monomers via the LIFO pathway more efficient than that via the FIFO pathway. On the other hand, when the first and the second halves of the DNA are equally rigid with ${K}_F = {K}_L$ (the dotted line in Figure 5C), we do not observe any difference in *E* between these two pathways.

As the difference in the rigidity (or the persistence length) between the first and the second halves of the DNA increases, a higher fraction of monomers ejects more efficiently via the LIFO pathway, i.e. *ϕ*_c_ becomes larger for LIFO than for FIFO. Figure 5D depicts the values of *ϕ*_c_ for the FIFO and LIFO pathways as a function of the relative rigidity $r\ = \ ( {{K}_F - {K}_L} )/{K}_L$. As $r$ increases, the ejection via the LIFO pathway becomes more efficient than that via the FIFO pathway. Because the value of $r$ of λ phage DNA in our study is approximately 0.13 (66), the difference in ${\phi }_c$ between LIFO and FIFO pathways in the simulations is expected to be approximately 9%, which is consistent with our experimental results: LamB 7.1%, glutaraldehyde 7.4%, 65°C 5.7% (Figure 4A). The differences in lengths observed for *in vitro* phage ejections and computer simulations provide additional evidence of both FIFO and LIFO mechanisms in addition to the sequence-specific staining results.

Single-molecule observations and computer simulations have shown that λ phage ejection can initiate from either end, which raises the question of why the FIFO and LIFO models have a nearly 50% probability for the LIFO model, rather than a 100% probability. One possible explanation is related to the process of phage infection, where λ phage particles first bind to the outer membrane protein LamB via the viral tail fiber before the phage DNA is ejected into the periplasm (72). The periplasmic nuclease NucA is known not to degrade λ DNA (73), and instead, mannose transporter complex in the inner membrane facilitates the entry of λ DNA into the cytoplasm. This complex process takes about 5 min *in vivo*, including pauses and stalls (74). In contrast, *in vitro* phage ejection occurs within a few seconds at a rate of 60 kb/s (71). During this process, a partial genome is present in the cytosol, and the first DNA to enter may have a higher likelihood of being attacked by restriction enzymes. Therefore, the exposure time of recognition sites for DNA-binding endonucleases may differ depending on which end of the DNA enters the cytoplasm first, as predicted by the FIFO and LIFO models.

Different *E. coli* strains have endonucleases with distinct DNA recognition sites. For instance, *Eco*RI (*E. coli* RY13), *Eco*72I (*E. coli* RFL72) and *Eco*110kI (*E. coli* BKM B-2004) cannot recognize the GC-rich region of the first half of λ DNA, whereas *Eco*143I (*E. coli* RFL143) and *Eco*105I (*E. coli* RFL105) cannot recognize the AT-rich region of the second half. Consequently, different endonucleases can degrade either the first or second half of λ DNA depending on the host strain. For instance, if λ phage infects the *E. coli* RY13 strain following the LIFO model, the last DNA end enters first and is degraded by *Eco*RI, while the FIFO model protects the DNA from degradation. Similarly, if λ phage infects other hosts such as the *E. coli* RFL143 strain, the LIFO model results in degradation by *Eco*143I, while the FIFO model protects the DNA. Therefore, the nearly 50% probability of ejecting the first or last DNA end is an effective survival strategy for λ phage to infect various *E. coli* strains.

Another notable aspect of *in vivo* phage ejection is the possibility of multiple bacteriophages infecting a host cell simultaneously (74). This combination of FIFO and LIFO from multiple viruses would further enhance the protection of the entire viral genome against restriction enzyme attack. With both ends of the DNA secure in different orientations, more proteins can be produced to construct mature bacteriophages. Other bacteriophages, such as T2 and T4, have circularly permuted genomes (75–77), which likely provide similar protection against restriction enzyme attack. Thus, the 50% probability of ejecting the first or last DNA end is an effective survival strategy for phage λ to infect various strains of *E. coli* as hosts. This bi-directional ejection allows the phage to evade detection and degradation by host restriction enzymes, as different parts of the genome are protected in different orientations.

In this study, we visualized phage-ejected DNA stained with AT-specific reagents. This visualization allowed us to determine the DNA directionality and the ejection lengths. Contrary to the popular model of only LIFO, single-molecule data and computer simulation results show that the DNA ejection of λ phages starts from either the left end or the right end. An interesting observation is that the ejected λ DNA length by FIFO is shorter than that by LIFO. We reproduced the same result through our computer simulations by considering GC-rich first-half and AT-rich second-half regions of λ DNA. The shorter length of the DNA ejected through the FIFO mechanism compared to the LIFO mechanism further supports this bi-directionality. Furthermore, the qPCR results obtained after DNase treatment of the ejected DNA also support the bi-directionality of the ejection process. The extremely condensed DNA within phage capsids is assumed to be immobile. However, our experiment and simulation studies demonstrate the possible mobility of the DNA with a liquid crystal density. This bi-directional ejection can be seen as a survival strategy for the phage, as it increases its chances of avoiding degradation by endonucleases present in its host cell. Additionally, this mobility of the DNA within the phage capsid, despite the extremely condensed state, highlights the potential importance of DNA mobility in viral survival and replication.

## DATA AVAILABILITY

The simulation codes and data underlying this article will be shared on reasonable request to the corresponding author.

## Supplementary Material

gkad340_Supplemental_Files
