# Supplementary material for: AT-specific DNA visualization revisits the directionality of bacteriophage λ DNA ejection
Source: Nucleic Acids Res. 2023 May 9;51(11):5634–46. doi: 10.1093/nar/gkad340 (PMC10287942; doi:10.1093/nar/gkad340)
Supplement: gkad340_Supplemental_Files [file gkad340_supplemental_files.zip › SI.pdf]

AT-specific DNA visualization revisits the directionality of bacteriophage λ DNA ejection

Serang Bong[1†], Chung Bin Park[1†], Shin-Gyu Cho[2†], Jaeyoung Bae[1†], Natalia Diyah Hapsari[1,3†], Xuelin Jin[1,4†], Sujung Heo[1], Ji-eun Lee[3], Kaori Hashiya[5], Toshikazu Bando[5], Hiroshi Sugiyama[5], Kwang-Hwan Jung[2,*], Bong June Sung[1,*], and Kyubong Jo[1,*]

[1] Department of Chemistry, Sogang University, Seoul, 04107, Korea
[2] Department of Life Science, Sogang University, Seoul, 04107, Korea
[3] Chemistry Education Program, Department of Mathematics and Science Education, Sanata Dharma University, Yogyakarta, 55282, Indonesia
[4] College of Agriculture, Yanbian University, Yanji, 133000, China.
[5] Department of Chemistry, Graduate School of Science, Kyoto University, Sakyo-Ku, Kyoto 606-8502, Japan

Supporting Information (SI)

Materials and Methods

- LamB Preparation
- Synthesis of H_2N-β_2-Py_4-β-Py_4-Dp (H_2N-P8).
- Synthesis of ATTO 647N-β_2-Py_4-β-Py_4-Dp (AP8)
- Binding Affinity Measurement between DNA and AP8 using FRET
- H-NS-mScarlet Preparation
- SDS-PAGE & Size Exclusion Chromatography
- Quantitative PCR for Analysis of Directionality of Ejection
- Observation of λ Phage Ejection Stained by YOYO-1 in Flow cell

Supplementary Figures and Movies

Figure S1. LamB purification.

Figure S2. ATTO 647N-P8

Figure S3. AP8 and TP8 fluorescence intensity comparison

Figure S4. FRET assay to measure the binding specificity of AP8 to DNA.

Figure S5. H-NS-mScarlet.

Figure S6. FRET assay to measure the binding specificity of H-NS-mScarlet to DNA

Figure S7. Quantitative PCR of the λ DNA remaining in the capsid after ejection and digestion

Movie S1. Real-time observation of phage ejection

Materials and Methods

LamB Preparation. SI Figure S1 illustrates the LamB (maltoporin) purification procedure (*Int J Biol Macromol*, **2006**, 39(1-3), 10-14, *Structure*, **2017**, 25(7), 1139). The *Lamb* gene (accession number - NP_418460.1) was isolated from the *Escherichia coli* UT5600 strain derived from the K-12 MG1655 strain. The gene was amplified by polymerase chain reaction (PCR) with two primers containing *Nde*I and *Xho*I restriction enzyme sites (ACTCCATATGATGATTACTCTGC GCAAAC and ACTCCTCGAGCCACCAGATTTCCATCTGG), and subsequently, the PCR fragment was introduced into the pET21b vector. The cloned plasmid was transformed into *E. coli* C43 (DE3), and then a single colony of the transformants was inoculated and incubated in LB containing 50 μg/mL ampicillin at 37°C and 200 rpm for 16 hours. The seed cultures were transferred 1:100 ratio to the volume of LB and further incubated at 37°C and 200 rpm until the OD_{600} reached 0.4~0.6. At this point, the cells were induced with 0.84 mM isopropyl β-D-1-thiogalactopyranoside (IPTG) and subsequently incubated for 4 hours. The induced cells were harvested and washed with buffer S (150 mM NaCl and 50 mM Tris-HCl at pH 7.0) by centrifugation at 5,000 g and 4°C for 20 min. The harvested cells were then resuspended with buffer S containing 1 mM Na_2-EDTA, 0.5 mM phenylmethylsulfonyl fluoride (PMSF), 10 μM leupeptin hemisulfate, and 1 μM pepstatin A. The resuspended cells were disrupted using a sonicator. The resulting cell lysates were then centrifuged at 3,000 g and 4°C for 20 min. The supernatants containing cell membranes were further isolated using ultracentrifugation (Beckman × L-90 ultracentrifuge, USA) at 100,000 × g and 4°C for an hour. The pellets containing the inner and outer membranes were solubilized with buffer S containing 1% *n*-dodecyl-β-D-maltopyranoside (DDM) at 4°C for 16 hours. Following inner membrane solubilization, the samples were then centrifuged at 45,000 x g and 4°C for 30 min. The pellets containing the outer membrane were solubilized with buffer S containing 3% *n*-octyl-β-maltopyranoside (OM) at 4°C for 16 hours. After outer membrane solubilization, insoluble proteins were removed by ultracentrifugation at 45,000 g and 4°C for 15 min. The supernatants were mixed with Ni^{2+} NTA agarose by rocking at 4°C for 4 hours. The LamB was washed twice with buffer S containing 1% OM and subsequently eluted with buffer S containing 1% OM and 250 mM imidazole. The buffer of purified LamB was exchanged three times with buffer NTE (150 mM NaCl, 40 mM Tris-acetate at pH 8.0, 1 mM Na_2-EDTA, and 1% OM) by Amicon Ultra-4 50,000 MWCO centrifugal filter units. The concentration of the purified LamB was quantified by the Bradford assay. Glycerol and dithiothreitol (DTT) were added to concentrated LamB (final composition - buffer NTE containing

30% glycerol, 1 mM DTT, and 250 μg/mL LamB) and subsequently stored at -20°C. LamB was stored at 10-fold concentration for further dilution.

Synthesis of H_2N-β$_2$-Py$_4$-β-Py$_4$-Dp (H_2N-P8). Fmoc solid-phase synthesis of H_2N-β$_2$-Py$_4$-β-Py$_4$-Dp was performed with a computer-assisted operation system as previously described (*Nucleic Acids Res.* **2018**, *46(18)*, e108). The workflow of the H_2N-P8 synthesis is shown in SI Figure S2. Fmoc units used in each step were Fmoc-Py-COOH (77 mg, 0.21 mmol) and Fmoc-β-COOH (66 mg, 0.21 mmol). Prior to solid-phase peptide synthesis (SPPS), each Fmoc unit was dissolved in NMP (1.0 mL) with HCTU, where the amount of HCTU for Fmoc-Py-COOH and Fmoc-β-COOH was 79 mg (0.19 mmol). The fmoc-Py-oxime resin was prepared with Fmoc-Py-COOH, oxime resin, HCTU, DIEA, and DMF based on the reported condition (*Bioorg. Med. Chem.* **2002**, *10*, 2767–2774). The resin was swelled with NMP and then loaded into the reaction vessel.

The following procedures were carried out for the synthesis of H_2N-β$_2$-Py$_4$-β-Py$_4$-Dp: Fmoc units were deblocked twice for 4 min with 20% piperidine/DMF (500 μL), followed by the addition of 10% DIEA/NMP (364 μL, with DIEA at 0.21 mmol) to the Fmoc unit-HCTU mixture for activation and coupling for 60 min. Each step included five washes with DMF. After the last coupling, the amino group at the N-terminal was deblocked. All couplings were performed using a single-coupling cycle. To stir the resin, all lines were purged with solution transfers and bubbled with nitrogen gas. Polypyrrole on the resin was cleaved with 1.0 mL 3-(dimethylamino) propylamine at 55 °C for 3 h after SPPS. The resin was removed by filtration and washed thoroughly with dichloromethane, and the filtrate was concentrated *in vacuo*. The residue was dissolved in 1.0–2.0 mL dichloromethane-methanol mixture, followed by the addition of over 10-fold volume of diethyl ether. The solution was centrifuged, and the supernatant was removed. This process was repeated until white precipitation was obtained. The crude product was purified using reversed-phase flash chromatography with a CombiFlash Rf (Teledyne Isco, Lincoln, NE) using a 4.3 g reversed-phase flash column (C18 RediSep Rf) in 0.1% TFA in water with acetonitrile as the eluent at a flow rate of 18.0 mL/min. The collected fractions were lyophilized to obtain the objective compound.

Synthesis of ATTO 647N-β$_2$-Py$_4$-β-Py$_4$-Dp (AP8). ATTO 647N NHS ester (1.0 mg, 1.2 × 10–3 mmol, Sigma-Aldrich) and H_2N-P8 (1.3 mg, 1.0 × 10–3 mmol) were dissolved in DMF (200 μL) and DIEA (0.50 μL, 2.4 × 10–3 mmol). After the reaction, the product was purified by reverse-phase HPLC followed by lyophilization of the collected fractions to afford AP8 as a blue powder.

Analytical HPLC: t_R = 16.3 min. MALDI–TOF–MS *m/z* calculated for $C_{104}H_{127}N_{24}O_{13}^+$ 1921.27 found 1921.685. (Figure S2)

Binding Affinity Measurement between DNA and AP8 using FRET. The binding affinity of AP8 to four 5'-6-FAM-modified DNA oligonucleotides containing 10/7/4/0-mer AT base pairs was measured using FRET (fluorescence resonance energy transfer).

The sequences of oligonucleotides were 5'-6-FAM-GGC CAT ATA TAT ATC CGG TTT TCC GGA TAT ATA TAT GGC C-3', 5'-6-FAM-GGC CGC ATA TAT AGC CGG TTT TCC GGC TAT ATA TGC GGC C-3', 5'-6-FAM-GGC CGC GAT ATG CGC CGG TTT TCC GGC GCA TAT CGC GGC C-3', 5'-6-FAM-GGC CGC GCG CGC GCC CGG TTT TCC GGG CGC GCG CGC GGC C-3' for 10, 7, 4, and 0-mer AT base pairs, respectively.

Each DNA oligonucleotide was diluted to 400 nM with 1× TE buffer and mixed with 4, 3.2, 2.4, 0.8, and 0.4 µM of AP8 at 1:1 volume ratio. After incubation at room temperature for at 30 min, fluorescence intensity was measured by Hitachi F-7000 fluorometer.

H-NS-mScarlet Preparation.

The plasmid of H-NS-mScarlet was constructed by combining two plasmids, H-NS-mCherry (*Analyst*, **2019**, 144(3), 921-927) and tTALE-mScarlet (*Molecules*, 2022, 27(16), 5248). To achieve this, the plasmid for tTALE-mScarlet in pET15b was first digested with restriction enzymes (*Nde*I & *Bam*HI) to remove tTALE-mScarlet. Then, the amplified H-NS gene was inserted and ligated into the plasmid using the AccuRapid Cloning Kit (Bioneer, Daejeon, Korea). This created the H-NS plasmid, which was used as the vector for H-NS-mScarlet. The amplified mScarlet gene was also inserted and ligated into the H-NS plasmid using the AccuRapid Cloning Kit (Bioneer, Daejeon, Korea). The primers used for amplifying the H-NS and mScarlet genes are described below.

H-NS forward primer: 5'-GCG GCC TGG TGC CGC GCG GCA GCC ATA TGA TGA GCG AAG CAC TTA AAA TTC TG-3'

H-NS reverse primer: 5'-CGG GCT TTG TTA GCA GCC GGA TCC TTG CTT GAT CAG GAA ATC GTC G-3'

mScarlet forward primer: 5'-CGA CGA TTT CCT GAT CAA GCA AGA ATT CAT GGT GAG CAA GGG CGA G-3'

mScarlet reverse primer: 5’-CTT TGT TAG CAG CCG GAT CCC TTG TAC AGC TCG TCC ATG CC

The amino acid sequence for H-NS-mScarlet is described below.

MGSSHHHHHHSSGLVPRGSHMMSEALKILNNIRTLRAQARECTLETLEEMLEKLEVVV
NERREEESAAAAEVEERTRKLQQYREMLIADGIDPNELLNSLAAVKSGTKAKRAQRPAK
YSYVDENGETKTWTGQGRTPAVIKKAMDEQGKSLDDFLIKQEFMVSKGEAVIKEFMRF
KVHMEGSMNGHEFEIEGEGEGRPYEGTQTAKLKVTKGGPLPFSWDILSPQFMYGSRAFT
KHPADIPDYYKQSFPEGFKWERVMNFEDGGAVTVTQDTSLEDGTLIYKVKLRGTNFPPD
GPVMQKKTMGWEASTERLYPEDGVLKGDIKMALRLKDGGRYLADFKTTYKAKKPVQ
MPGAYNVDRKLDITSHNEDYTVVEQYERSEGRHSTGGMDELYKGSGC (44.64 KDa)

The constructed H-NS-mScarlet plasmid was transformed into *E. coli* BL21 (DE3) strains using a standard cloning procedure. A single colony of the transformed cells was inoculated in fresh LB media containing ampicillin and incubated for 1 hr until fully saturated. A subsequent culture was conducted until the optical density reached ~0.6 at 37 °C with corresponding antibiotics. H-NS-mScarlet was induced overnight using 1 mM IPTG in a shaker at 20°C and 200 rpm. Cells for protein purification were harvested by centrifugation at 10,000g for 10 min, and the residual media was washed using cell lysis buffer (50 mM Na_2HPO_4, 300 mM NaCl, 10 mM Imidazole, pH 8.0). The cells were lysed by ultrasonication for 15 min, and the cell debris was centrifuged at 10,000 rpm for 10 min at 4°C. His-tagged H-NS-mScarlet proteins were purified using affinity chromatography with Ni-NTA agarose resin. For this affinity chromatography, the protein-resin mixture was kept on a shaking platform at 4 °C for 2 hrs. The lysate containing proteins bound to Ni-NTA agarose resin was loaded onto the column for gravity chromatography and rinsed several times using protein washing buffer (50 mM Na_2HPO_4, 300 mM NaCl, 20 mM Imidazole, pH 8.0). Finally, the proteins were eluted using protein elution buffer (50 mM Na_2HPO_4, 300 mM NaCl, 250 mM imidazole, pH 8.0). All proteins were diluted (10 μg/mL) using 50 % w/w glycerol/1×TE buffer (Tris 10 mM, EDTA 1 mM, pH 8.0).

SDS-PAGE & Size Exclusion Chromatography

SI Figure S5 (C) displays the results of SDS-PAGE analysis of the purified H-NS-mScarlet protein, which has a molecular weight of 44.64 kDa. The distribution of oligomerization states of the protein was also analysed using size exclusion chromatography, and it was found that it primarily consists of monomer, dimers and trimers, as shown in SI Figure S5 (D). The molecular weights

were determined by comparing them to the SEC MW standards (GE Healthcare, Chicago, USA). The column was a Superdex® 200 Increase 10/300 GL column with a phosphate buffered saline buffer (pH 8.0).

Quantitative PCR for Analysis of directionality of ejection

To induce DNA ejection by heat, 20 μL of phage λ stock solution was incubated at 65°C for 5 min. To obtain the remaining DNA inside the capsids, 5 U of DNase I (Enzynomics) was added to the solution and incubated at 37°C for 30 min in 1× DNase I buffer, followed by inactivation at 75°C for 20 min in the presence of 5 mM EDTA. For the ejection by LamB and glutaraldehyde, λ phage was mixed with molten 2% LMP agarose (Thermo) solution for 0.7%, dispensed as a 20 μL droplet, and solidified at 4 °C. λ phage plugs were treated with 25 μg/mL of LamB or 2.5% glutaraldehyde. After ejection, the plugs were washed with 1× TM buffer. Then, 20 U of DNase I was added and incubated for 1 h at 37°C, followed by inactivation at 75 °C for 20 min in the presence of 5 mM EDTA. Molten agarose was cooled to 42 °C and incubated with 1 U of β-agarase for 1 h. The β-agarase I was then inactivated at 65 °C for 15 min. For qPCR analysis, 1 μL of reaction mixtures were used as the template. Five sets of primers were designed to amplify 300 bp region of 0.5, 12.1, 24.1, 36.2, and 47.7 kb of λ DNA. The primer sequences were as follows: TCG ACT TAC GCG TGC GC/AAT GCC GCA GCC TGT TAA CC for 451-750 bp, CTG GCG CAT AAA GAT GAG ACG C/ CGC CCA GGT CTT TTT CTG CTC for 12126-12425 bp, TAG AGT TGT GGC TTG GCT CTG C/ACC AAT TCC TAG GCA GGT CAT TGG for 24109-24408 bp, GTG GCG GAA AAG GAG ATA GCA AAT G/ TCA GAC ATT AGC CTG CGG GC for 36176-36475 bp, and CCG CTA GAT GAA GAG CAA GCG/GAC TGC GTG TTG GCT CTG TC for 47703 to 48002 bp. qPCR was performed with Exicycler 96 (Bioneer) using the following conditions: 5 min at 95 °C, followed by 40 cycles of 15 s at 95 °C, 30 s at 55 °C, and 30 s at 72 °C. Control experiments were conducted using 5 ng, 500 pg, 50 pg, and 5 pg of λ DNA (NEB) in 20 μL as the template.

Observation of λ phage ejection stained by YOYO-1 in flow cell

To visualize the ejection of λ DNA using glutaraldehyde, bacteriophage λ in TM buffer (50 mM Tris, 10 mM $MgCl_2$, pH 8.0) was loaded into flow cell and immobilized on neutravidin-coated surface. 4 U of DNase I was then treated with 1× DNase I reaction buffer to degrade prematurely released λ DNA. After 30 min of incubation at 37 °C, the flow cell was washed with at least 200 μL of 1× TE buffer to remove residual DNase I. 50 μL of YOYO-1 in TM buffer with 4% β-

mercaptoethanol was loaded into the chamber to stain λ DNA in capsid. To eject DNA, 2.5% glutaraldehyde solution in 0.2 M sodium phosphate buffer (pH 7.25) was loaded. (Movie S1)

Supplementary Figures and Movie

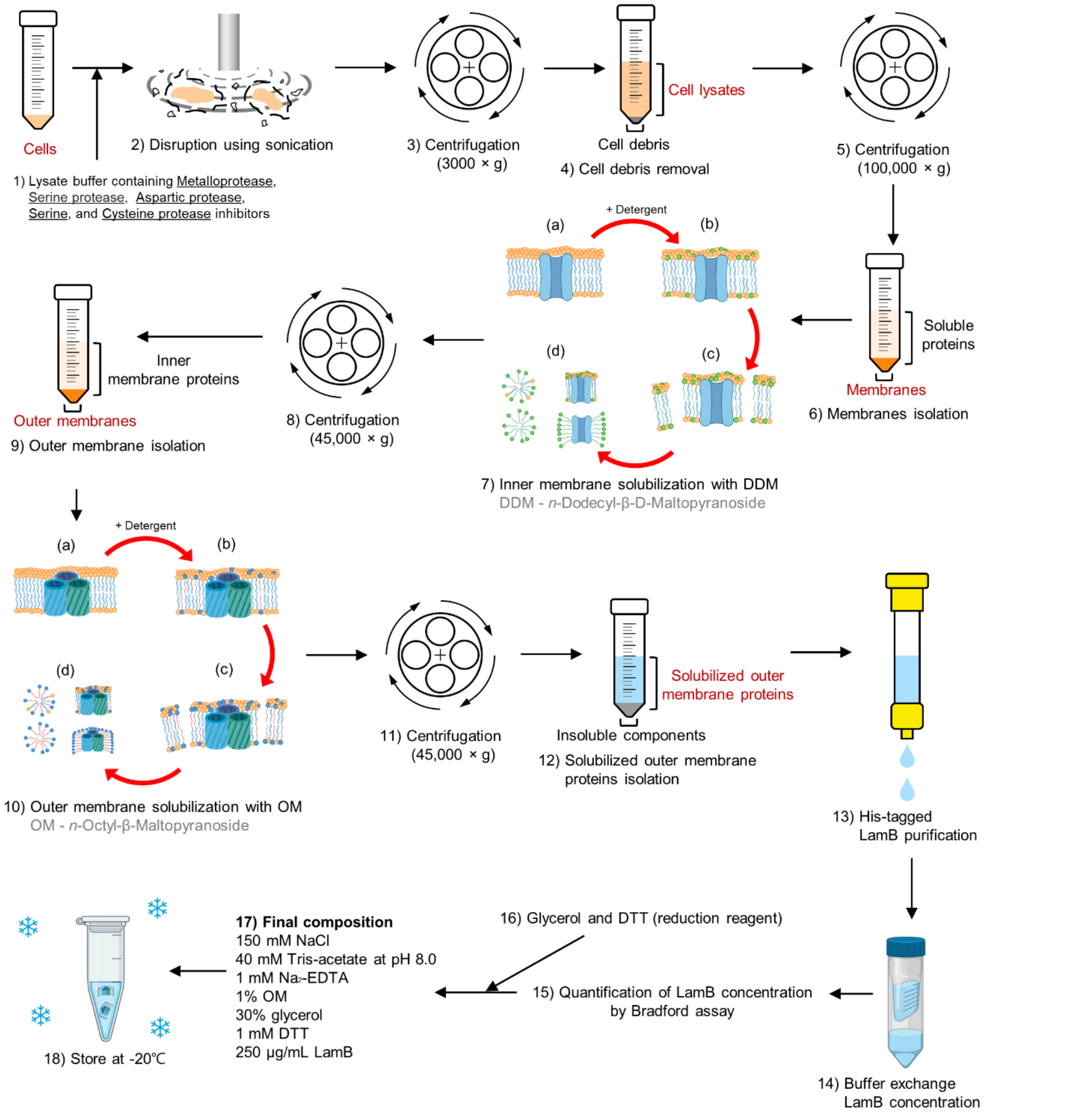

Figure S1. LamB purification. Steps 1-4: isolation of soluble proteins and membranes. **Steps 5-6:** isolation of the membrane. **Steps 7-9:** isolation of the outer membrane. **Steps 10-12:** isolation of solubilized outer membrane proteins. **Step 13:** His-tag protein purification with affinity chromatography. **Step 14:** Removal of imidazole using elution buffer. **Steps 15-18:** Preparation of the composition for storage. Samples to be used in the next step are highlighted in red. (©Biorender-biorender.com).

(A)

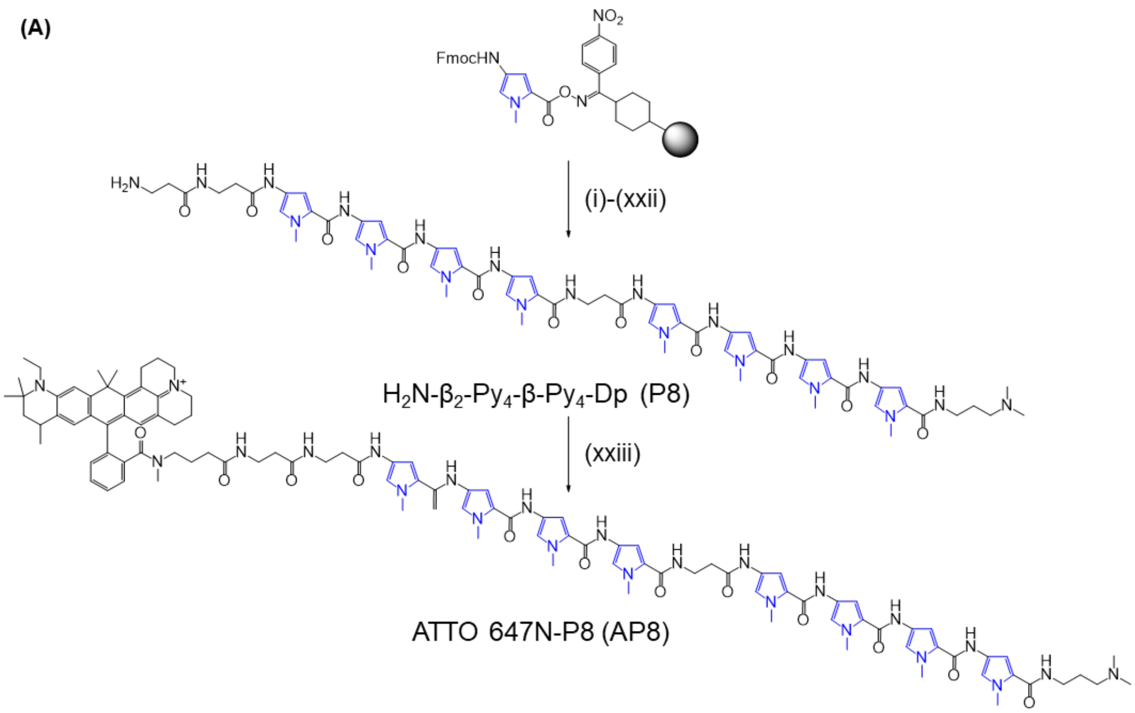

(B)

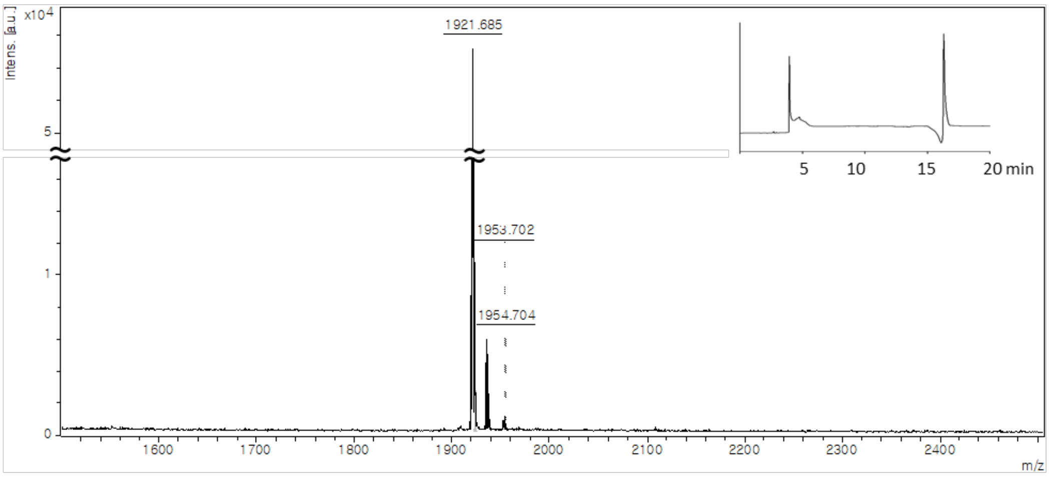

Figure S2. ATTO 647N-P8 (AP8). (A) ATTO 647N-P8 was synthesized using the following steps: (i) 20% Piperidine, DMF; (ii) Fmoc-Py-COOH, HCTU, DIEA, NMP; (iii) 20% Piperidine, DMF; (iv) Fmoc-Py-COOH, HCTU, DIEA, NMP; (v) 20% Piperidine, DMF; (vi) Fmoc-Py-COOH, HCTU, DIEA, NMP; (vii) 20% Piperidine, DMF; (viii) Fmoc-β-Ala-COOH, HCTU, DIEA, NMP; (ix) 20% Piperidine, DMF; (x) Fmoc-Py-COOH, HCTU, DIEA, NMP; (xi) 20% Piperidine, DMF; (xii) Fmoc-Py-COOH, HCTU, DIEA, NMP; (xiii) 20% Piperidine, DMF; (xiv) Fmoc-Py-COOH, HCTU, DIEA, NMP; (xv) 20% Piperidine, DMF; (xvi) Fmoc-Py-COOH, HCTU, DIEA, NMP; (xvii) 20% Piperidine, DMF; (xviii) Fmoc-β-Ala-COOH, HCTU, DIEA, NMP; (xix) 20% Piperidine, DMF; (xx) Fmoc-β-Ala-COOH, HCTU, DIEA, NMP; (xxi) 20% Piperidine, DMF; (xxii) 3-(Dimethylamino) propylamine, 55 °C, 3 h; (xxiii) ATTO 647N NHS ester, DIEA, DMF. (B) MALDI-TOF-MS spectrum of ATTO 647N-β$_2$-Py$_4$-β-Py$_4$-Dp after HPLC purification at the peak of 16.3 min, as shown in the inset chromatogram (647 nm). The gradient changed from 60:40 (acetonitrile:0.1% trifluoroacetic acid in water) to 50:50.

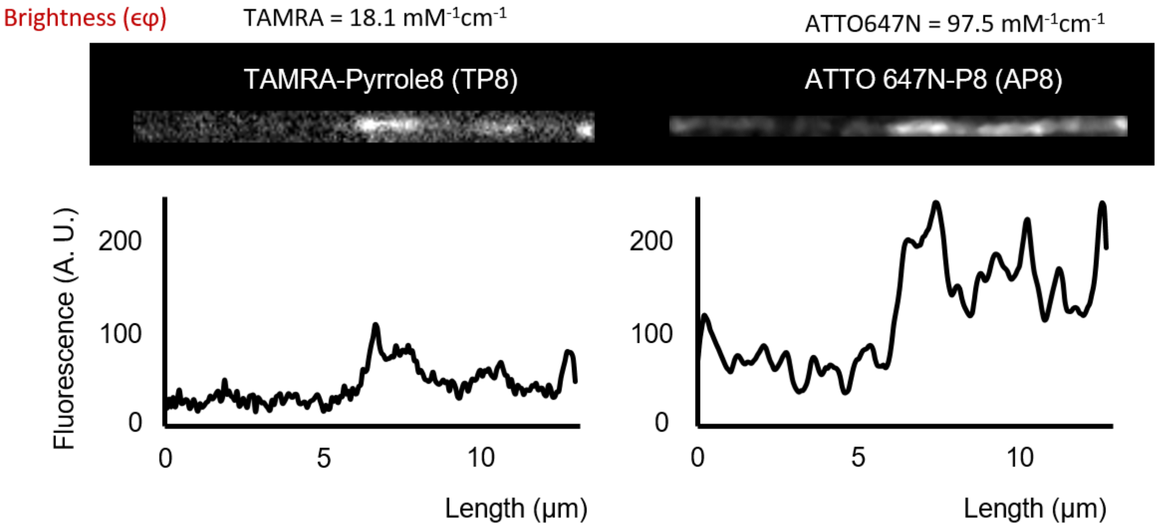

Figure S3. **AP8 and TP8 fluorescence intensity comparison**: Comparison of fluorescent intensities for TAMRA-β$_2$-Py$_4$-β-Py$_4$-Dp (TP8) and ATTO 647N-β$_2$-Py$_4$-β-Py$_4$-Dp (AP8) stained bacteriophage λ DNA. The images were captured using a 16-bit TIFF format (65,536 grey levels) by the Photometrics PRIME camera with an exposure time of 100 ms.

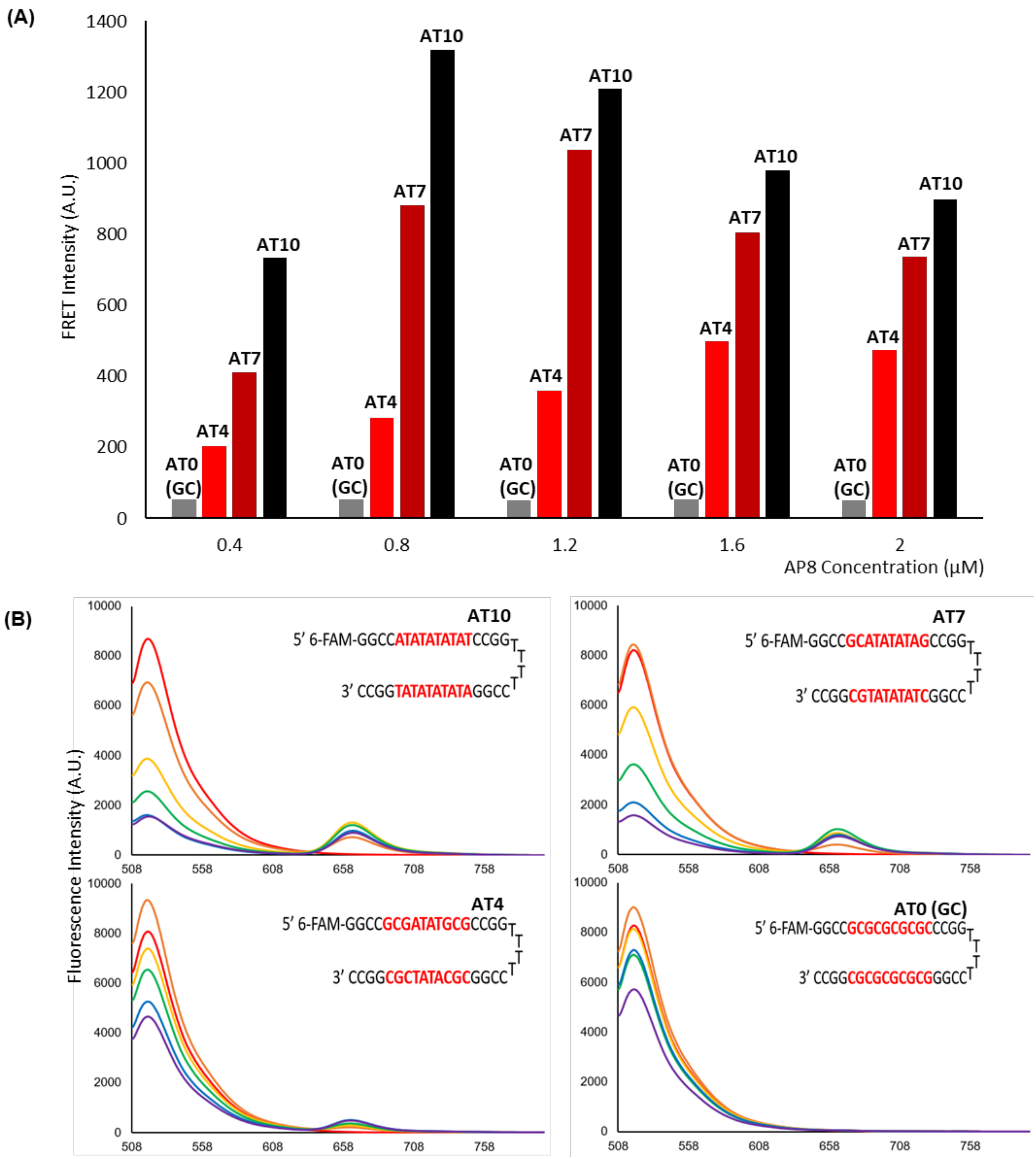

Figure S4. FRET assay to measure the binding specificity of AP8 to DNA. (A) Fluorescence intensity changes of AP8 bound to 5'-6-FAM-oligonucleotides at 664 nm, with respect to the length of AT base pairs. Oligonucleotides with no AT base pairs showes no FRET signal, whereas longer AT base pairs show higher FRET intensities. (B) Emission spectra of 5'-6-FAM-oligonucleotides with different concentrations of AP8 (2 (red)/1.6 (orange)/1.2 (yellow)/0.8 (green)/0.4 (blue)/0 (purple) μM).

(A) H-NS-mScarlet sequence

```
MGSSHHHHHHSSGLVPRGSHMMSEALKILNNIRTLRAQARECTLETLEEMLEKLEVVVNERREEESAAAAEVEERTRKLQQYR
EMLIADGIDPNELLNSLAAVKSGTKAKRAQRPAKYSYVDENGETKTWTGQGRTPAVIKKAMDEQGKSLDDFLIKQEFMVSKGE
AVIKEFMRFKVHMEGSMNGHEFEIEGEGEGRPYEGTQTAKLKVTKGGPLPFSWDILSPQFMYGSRAFTKHPADIPDYYKQSFP
EGFKWERVMNFEDGGAVTVTQDTSLEDGTLIYKVKLRGTNFPPDGPVMQKKTMGWEASTERLYPEDGVLKGDIKMALRLKDGG
RYLADFKTTYKAKKPVQMPGAYNVDRKLDITSHNEDYTVVEQYERSEGRHSTGGMDELYKGSGC(44.64 KDa)
```

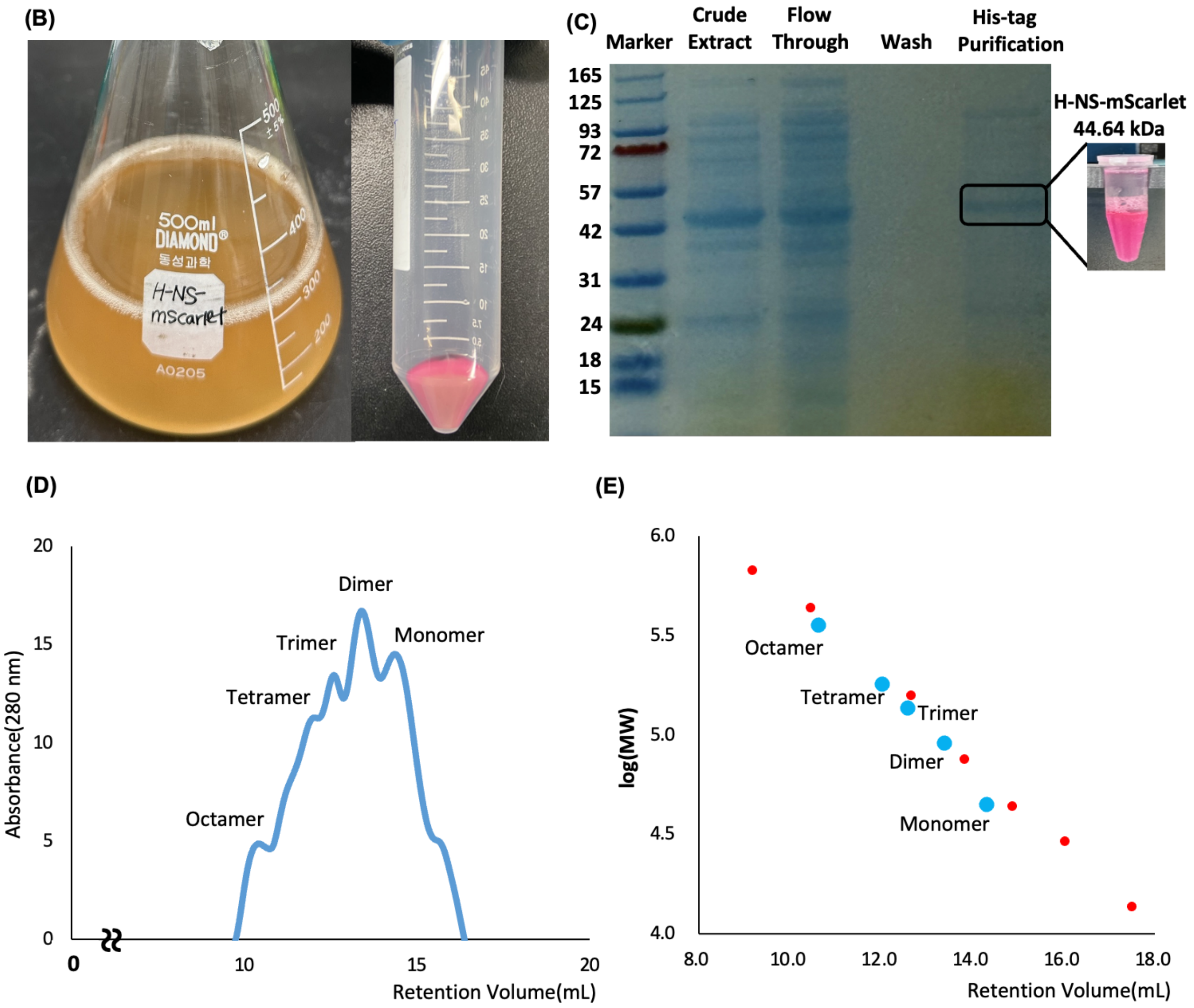

Figure S5. H-NS-mScarlet. (A) The sequence of H-NS-mScarlet. (B) Expression and harvesting of H-NS-mScarlet. (C) SDS-PAGE results for affinity chromatography purification of H-NS-mScarlet (44.64 kDa) and a photo of the purified H-NS-mScarlet. (D) Size exclusion chromatography of H-NS-mScarlet. (E) The graph shows the linear relationship between log(MW) and retention volume. MW stands for molecular weight. The blue circles represent H-NS-mScarlet, and the red circles represent MW standards: 13.7, 29, 44, 75, 158, 440, 669 kDa.

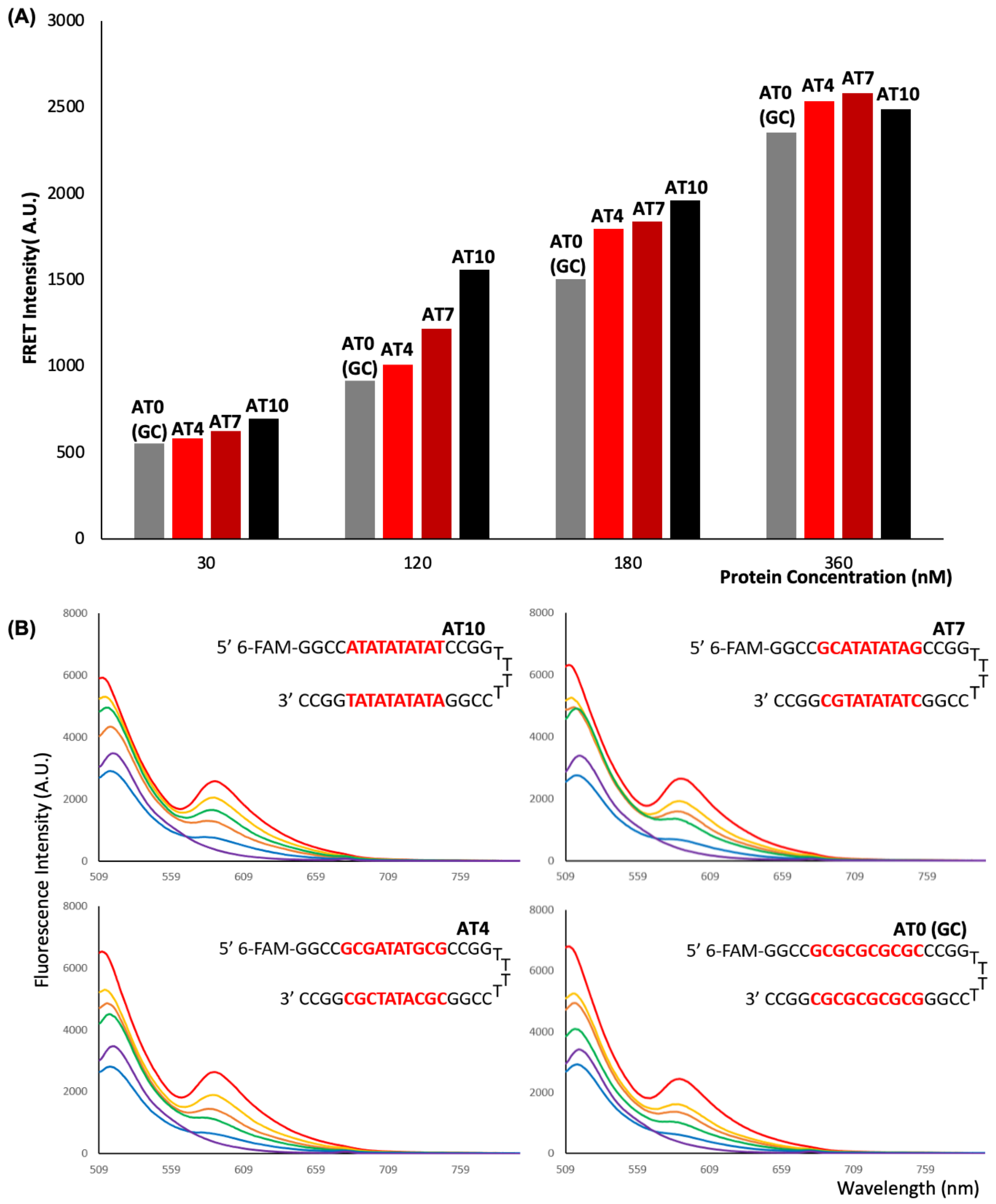

Figure S6. FRET assay to measure the binding specificity of H-NS-mScarlet to DNA. (A) Fluorescence intensity changes of H-NS-mScarlet bound to 5'-6-FAM-oligonucleotides at 594 nm, with respect to the length of AT base pairs (AT0, AT4, AT7 and AT10). (B) Emission spectra of 5'-6-FAM-oligonucleotides with different concentrations of H-NS-mScarlet (360 (red)/240 (orange)/180 (yellow)/120 (green)/30 (blue)/0 (purple) nM).

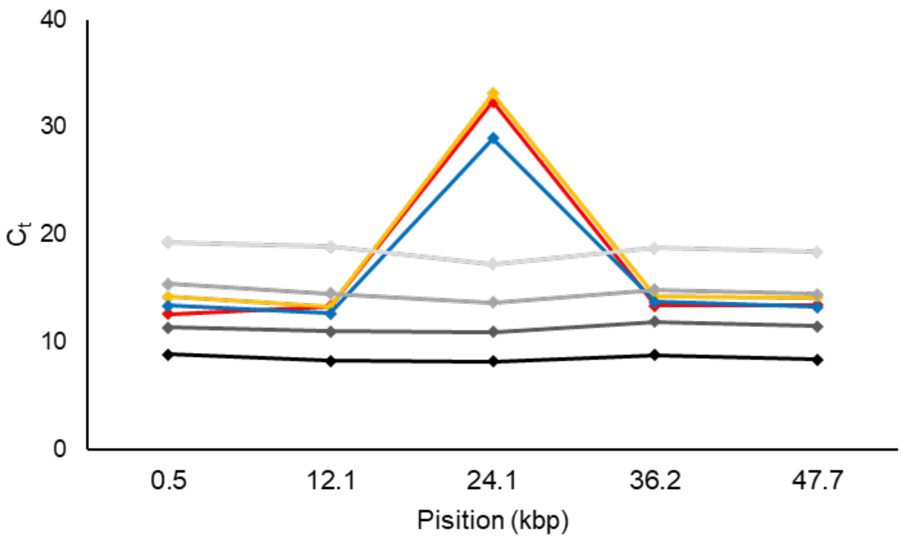

Figure S7. Quantitative PCR of the λ DNA remaining in the capsid after ejection and digestion. Bacteriophage λ was ejected by three induction methods (◆: heat, ◆: glutaraldehyde, ◆: LamB.) Then, ejected DNA was digested by DNase. The DNA remaining in the capsid waw quantified by qPCR at five positions (0.5, 12.1, 24.1, 36.2 and 47.7 kb). The amplicon at the centre (24.1 kb) is less abundant than the other positions. This result supports the notion that the ejection of λ phage is bidirectional. Control λ DNA (NEB): ◆: 5 ng, ◆: 500 pg, ◆: 50 pg, and ◆: 5 pg in 20 μL.

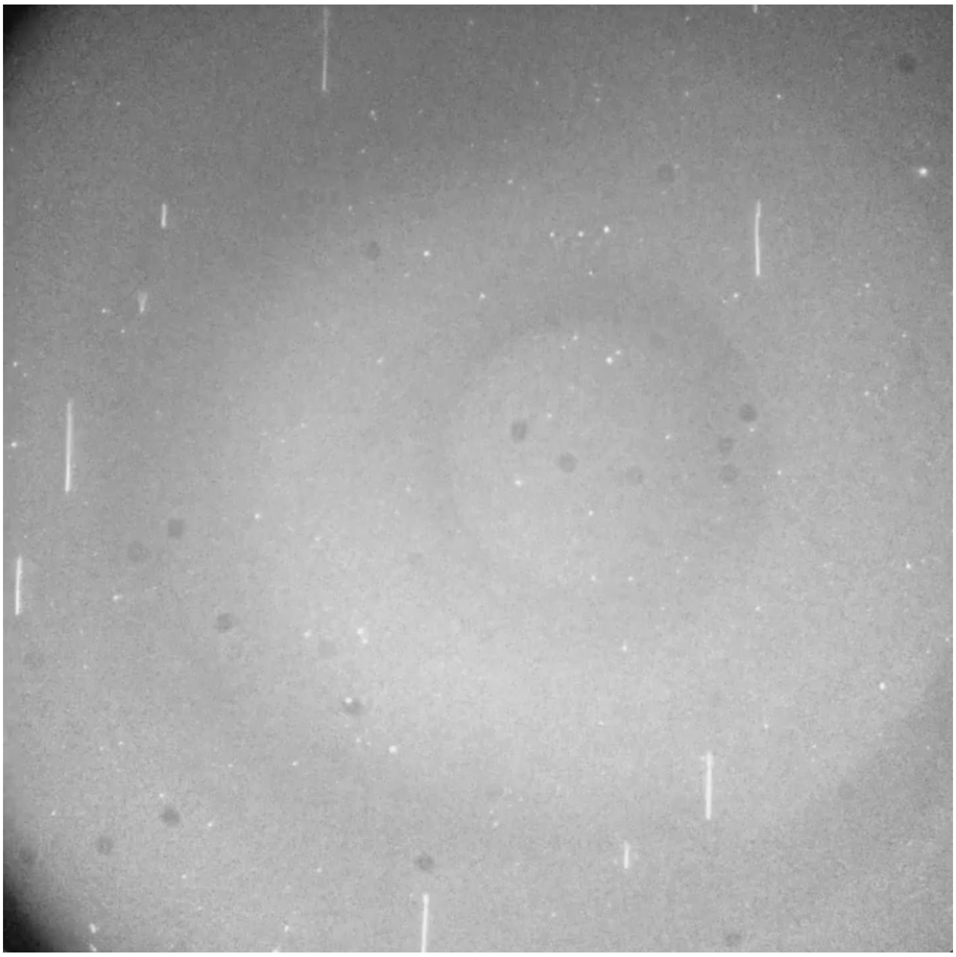

SI Movie S1. Real-time observation of phage ejection. YOYO-1 was added before the phage ejection process because it can penetrate the phage capsid. The ejection was induced by glutaraldehyde.
